# Heat Stress‐Mediated Multi‐Organ Injury: Pathophysiology and Treatment Strategies

**DOI:** 10.1002/cph4.70012

**Published:** 2025-05-14

**Authors:** Xiaoqing Ding, Binghong Gao

**Affiliations:** ^1^ School of Athletic Performance Shanghai University of Sport Shanghai China; ^2^ Faculty of Health Sciences and Sports Macao Polytechnic University Macao China

**Keywords:** apoptosis, heat stress, inflammation, mitochondria, multi‐organ injury, oxidative stress

## Abstract

With global warming in recent years, humans have been subjected to the impact of thermal environments on both work and life. Improving the body's ability to withstand heat is an urgent task. Accordingly, we summarize the signaling pathways in response to heat and the effects of heat stress on related physiological processes, such as mitochondrial health, inflammation, oxidative stress, and apoptosis, which provide a theoretical basis for understanding the damaging effects of a thermal environment. Based on this, we further summarize the multi‐organ injury caused by heat stress and its specific pathophysiological mechanisms to provide advice on coping strategies for people who need to perform physical activity or sports in a hot environment. In summary, this study provides research targets for future clinical research and ideas for practical application by summarizing the physiological and pathological processes and current coping strategies under heat stress.

## Graphical abstract

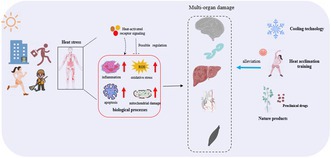

## 1 Introduction

Humans are homeotherms. Although a certain degree of fluctuation in body temperature occurs due to sex, age, activity intensity, disease, etc., the overall body temperature is relatively stable because of the ability of the human body to regulate heat production and heat dissipation to maintain a relative balance between the two, presenting a remarkable ability to adapt to heat stress. In recent years, high temperatures have become a public health threat, with surface temperatures as high as 40°C to 46°C recorded in some countries (The L.P.H. 2022). As a result, humans are subjected to thermal environmental shocks in both work and life, especially athletes who participate in competitive sports activities in thermal environments. With increased participation in sports events, avoiding physical activities or sports, even competitive sports, during high temperatures is impossible; therefore, some countries must develop heat action plans (Guardaro 2023). In addition to environmental planning and management, how to improve the tolerance of the human body to thermal environments and reduce the damage caused by high temperatures is currently a hot topic.

Heat is inevitable and can cause damage to the human body. How to understand the mechanisms of heat damage and how to mitigate damage from heat deserves attention. Environmental temperature changes affect many properties and functions of cellular biomolecules and structural components, such as protein activity, lipid structure and rigidity, and cell membrane fluidity and permeability (Somero 1995). When an organism is acutely exposed to a thermal environment, a series of stress responses are produced, manifested by increased core, skin, and brain temperatures, increased cardiovascular strain, and a greater reliance on carbohydrate metabolism, ultimately leading to a decrease in the aerobic performance of the organism (Febbraio et al. 1985; Périard et al. 2015). Mechanistically, this acute response of the organism may be attributed to cytoskeletal defects, increased membrane permeability, aggregation of ribosomal proteins, deposition of large numbers of particles in the cytoplasm, decreased intracellular pH, and cell cycle arrest due to the disruption of ionic homeostasis in response to heat stress. The heat response signals belong to the heat shock protein (HSP) family; however, a breakthrough in the ability of TRPV1 to respond to thermal stimuli has led to a new approach to understanding cellular responses to thermal environments, providing a molecular target for studying how cells generate a robust transcriptional response in thermal environments. The cellular response to thermal environments involves changes in metabolic levels, signaling pathways, biochemical responses, and different homeostatic changes to ensure cell survival in thermal environments. However, when the core temperature of the body is continuously increased by thermal stimulation, endothelial cell function is vulnerable to damage, leading to capillary leakage, imbalance of intracellular and extracellular environments, and release of inflammatory mediators, which will trigger pathophysiological processes with systemic effects. At the same time, extensive endothelial cell damage will affect the function of various organ systems and may further lead to multiple organ failure through the systemic inflammatory response. The severity and prognosis of this failure depend on the severity of the condition, the timeliness and precision of treatment, and the underlying health of the patient. However, the biological processes that occur in the body during heat stress and the mechanism of multiple organ damage caused by heat stress are still unclear.

**FIGURE 1:**
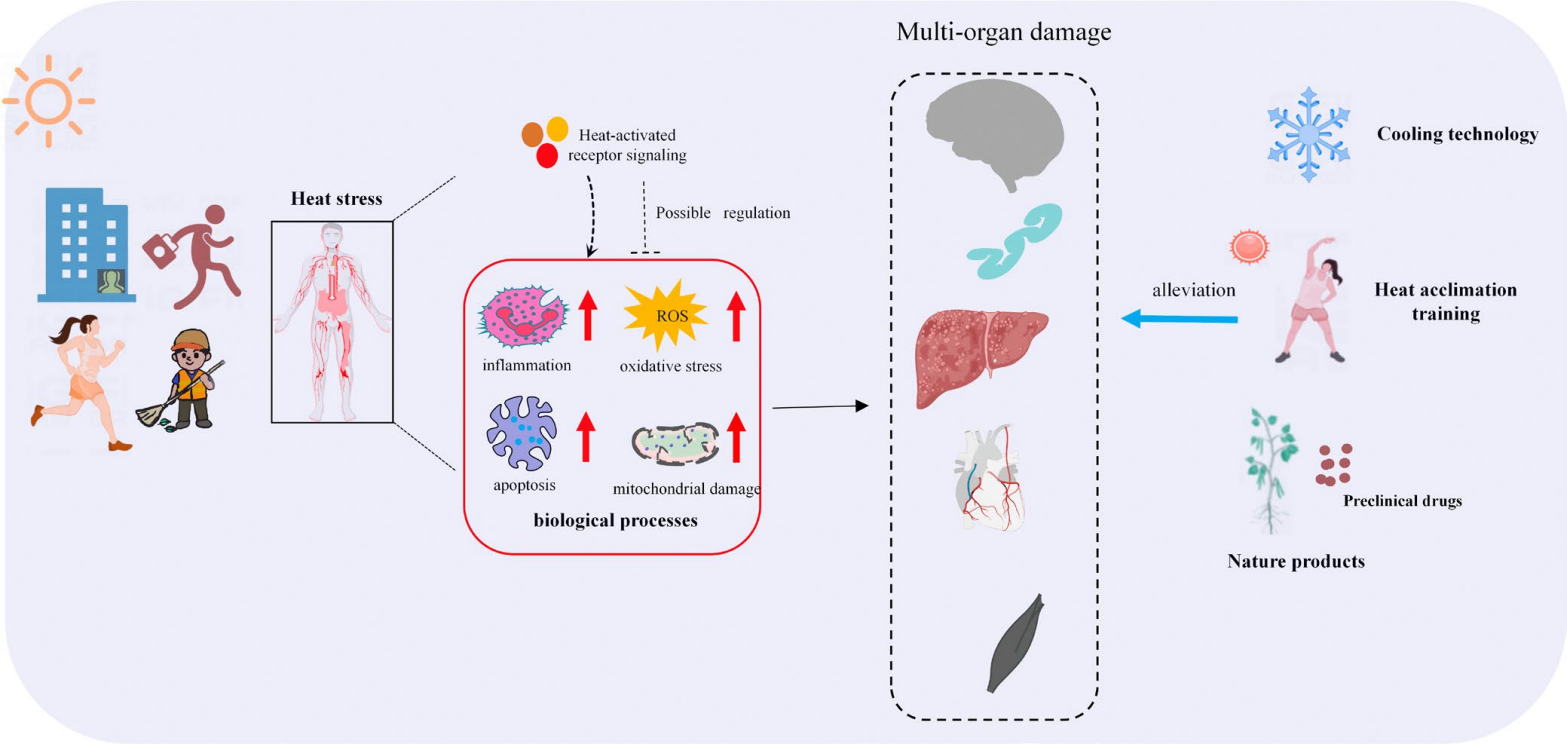
Thermal environment‐mediated pathophysiologic processes in the body and their coping strategies. Working or exercising in extreme heat environments can lead to heat stress in the organism. Heat stress can induce inflammatory responses, oxidative stress, apoptosis, and mitochondrial damage in the body by activating heat‐sensitive signaling pathways, leading to multi‐organ injuries such as central nervous system injury, intestinal dysfunction, liver injury, cardiovascular system injury, and skeletal muscle injury. Current coping strategies for heat stress injury include cooling techniques, heat acclimatization training, and the use of preclinical medications and natural products, and these coping strategies can mitigate a range of pathological processes induced by heat stress, thereby reducing multi‐organ damage.

Therefore, in this review, we first discuss current information on how cells respond to thermal environments, such as relevant transduction signals in thermal environments and a range of physiological responses. Then, this article further reviews the physiopathological mechanisms of multi‐organ injury under heat stress to provide more strategies for the prevention and treatment of heat injury (Figure 1).

## 2 Thermal Environment‐Mediated Biological Processes

**FIGURE 2:**
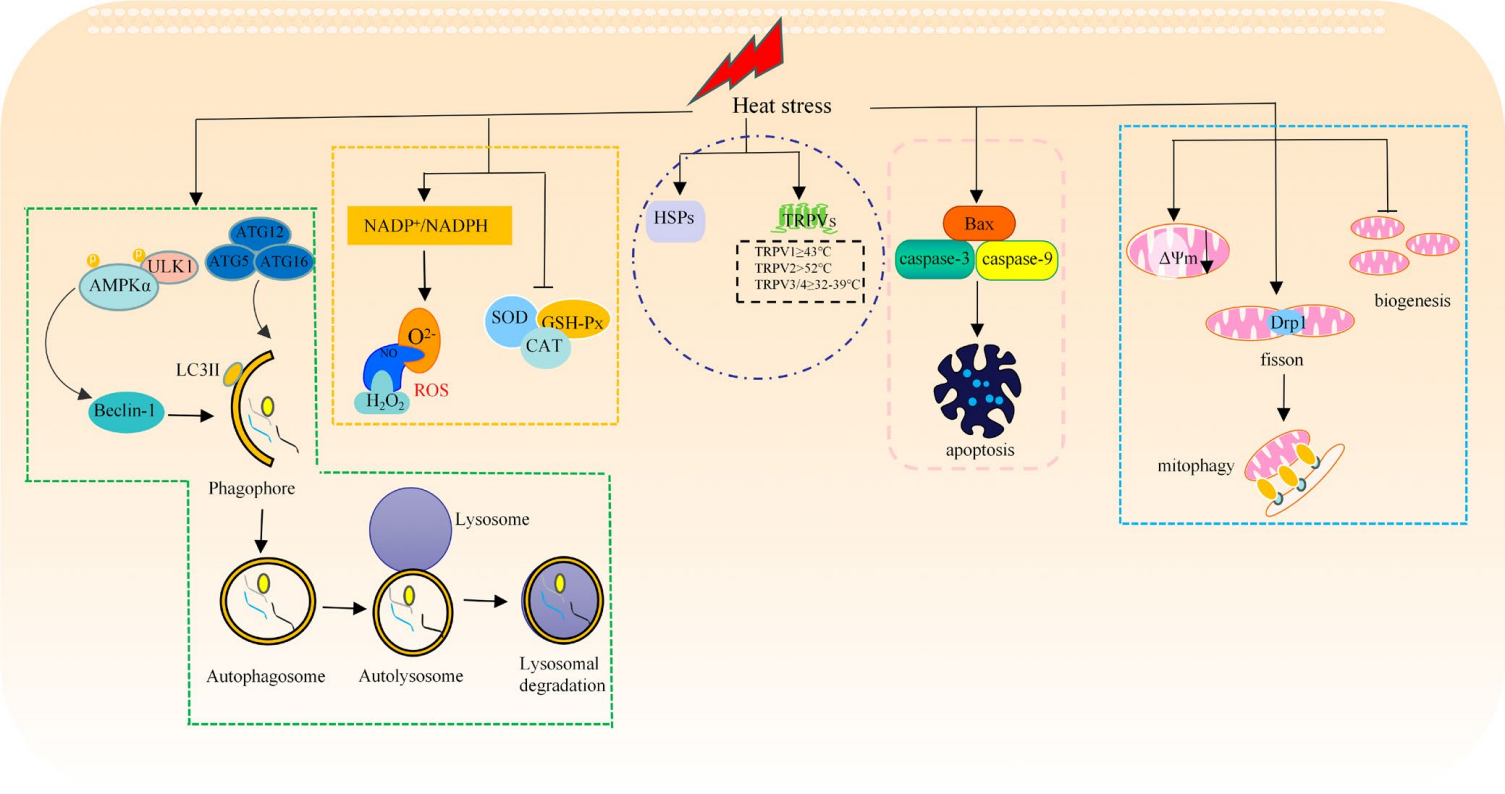
Heat stress can induce changes in biological processes such as heat signaling response, oxidative stress, mitochondrial damage, autophagy and apoptosis. Heat stress activates HSPs and TRPV signaling and induces heat‐responsive signaling. Heat stress increases the NADP^+^/NADPH ratio, promotes the expression of O^2−^, H_2_O_2_, and NO, and decreases the expression of SOD, GSH‐Px, and CAT, promotes ROS production, and disrupts antioxidant defenses to cause oxidative stress. In addition, under heat stress, protective autophagy was activated to prevent cellular dysfunction caused by accumulation of damaged organelles and proteins during heat stress. In addition, heat stress promotes apoptosis by activating the Bax, caspase signaling cascade. Heat stress also decreases ΔΨm, inhibits mitochondrial biogenesis and synthesis, and is accompanied by a stress‐induced increase in mitochondrial autophagy levels.

Heat stress is the sum of nonspecific responses to excessively high temperatures that exceed the ability of humans or animals to self‐regulate their body temperature (Bianca 1962). A thermoneutral zone exists in all theropods, and when the ambient temperature is higher than the upper limit of the thermoneutral zone, heat stress develops, leading to a series of physiological or pathological response reactions, such as thermal signaling reactions, oxidative stress, mitochondrial damage, autophagy, and apoptosis (Dantzer and Mormède 1983) (Figure 2).

### 2.1 Heat‐Signaling Response

#### 2.1.1 HSP

HSP is highly conserved among species and has a molecular weight range of 8–110 kDa (Jakob et al. 1999; Richter et al. 2010; Jee 2016). The family of HSPs includes HSP27 (small HSP, HSPB), HSP40 (DNAJ), HSP60, HSP70 (HSPA), HSP90 (HSPC), and HSP110/104 (HSPH) (Kregel 1985). High‐molecular‐weight HSPs are ATP‐dependent chaperone proteins, whereas small‐molecule HSPs act in an ATP‐independent manner. Heat shock proteins are protective proteins. In the absence of stress, chaperonins promote signaling and protein transport. When cells are stimulated by external stimuli (e.g., high temperature, hypoxia, chemical stimuli), it may lead to apoptosis or even cell death. At this time, heat shock proteins are transiently over‐synthesized. This defense mechanism is called the heat shock response (Kim et al. 2020). HSP regulates programmed cell death by eliminating stress responses to degrade protein misfolding and aberrant aggregation and to manage protein fragments (Schmitt et al. 2007). However, excess heat shock proteins may inhibit cell proliferation, which can cause several adverse effects (Zhang et al. 2020). Most heat shock proteins are heat‐induced, and they are transiently up‐regulated in response to heat stress, thereby maintaining cellular homeostasis (Dubińska‐Magiera et al. 2014). The specific mechanism may be that they play a key regulatory role in heat stress by stabilizing protein conformation or helping protein aggregation and dispersion (Zhang and Yu 2022; Bastaki et al. 2023). Thus, HSPs have the potential to be an early warning indication of the onset of heat stress in the organism.

#### 2.1.2 Thermo‐TRPs

The ability to sense external temperature is assumed by somatosensory neurons, which consist of a diverse population of genes with specific expression, including thermo‐TRPs, that are responsible for thermal conduction over a wide range of temperatures in the peripheral terminals of somatosensory neurons, whereby temperature information is converted into neural activity by afferents to the central nervous system (CNS) (Usoskin et al. 2015).

According to the degree of heat perception, thermo‐TRPs can be classified into noxious and non‐noxious thermoreceptors. TRP cation channel subfamily V member 1 (TRPV1) is one of the founding members of the mammalian thermal TRP family, which is expressed predominantly in small‐diameter neurons in the sensory ganglia and responds to noxious hyperthermia (> 43°C) (Bohlen et al. 2010). Under inflammatory conditions, the temperature sensitivity of TRPV1 is elevated, and the temperature threshold for activation is lowered. This leads to activation of TRPV1 at normal body temperature, which induces inflammatory pain (Moriyama et al. 2005). TRP subfamily M member 3 (TRPM3), which is also involved in noxious heat sensing, is highly co‐expressed with TRPV1 in small‐diameter sensory neurons, and its activity increases with temperature. TRPM3 knockout mice have impaired heat sensitivity, but behavioral responses to noxious heat stimuli are unaffected (Vriens et al. 2011). In addition, TRPV2 is expressed in sensory neurons, where it is capable of sensing temperature changes above 52°C (Shibasaki et al. 2010).

TRPV3 and TRPV4 are homologous channels involved in the perception of harmless warmth. Where TRPV3 has an activation threshold of 32°C–39°C, TRPV4 is activated between 25°C and 34°C (Güler et al. 2002). In terms of thermal gradients, wild‐type mice were unable to distinguish between floor temperatures of 30°C and 34°C, whereas TRPV4 knockout mice showed a strong preference for 34°C. The TRPV4 knockout mice also showed a defect in inflammation‐induced thermal hyperalgesia. In addition, TRPV4 knockout mice showed deficits in inflammation‐induced thermal nociceptive sensitization, and TRPV4 knockout mice exhibited a prolonged withdrawal latency during acute tail heating (Lee et al. 2005). TRPM2 is an innocuous thermoreceptor protein expressed mainly in non‐neuronal cells, and its temperature sensitivity is regulated by metabolic factors such as redox signaling in the body temperature range (Kashio 2021; Kashio et al. 2012). TRPM2 knockout mice showed an inability to perceive the temperature difference between 32°C and 38°C, whereas wild‐type mice preferred 33°C (Kashio et al. 2012). Thus, the response of thermo‐TRPs to temperature is important and clear.

### 2.2 Heat Stress and Oxidative Stress

Oxidative stress is caused by an imbalance in reactive oxygen species (ROS) production and scavenging capacity of the mitochondria (Luo et al. 2020). ROS are important signaling molecules during oxidative stress and mainly include superoxide anions (O^2−^), hydrogen peroxide (H_2_O_2_), nitric oxide (NO), and peroxynitrite (ONOO^−^). Small concentrations of ROS are required for cell signaling; however, when the concentration of ROS is uncontrolled, an oxygen radical‐mediated chain reaction is induced. This reaction causes failure of the intracellular antioxidant defense system, which comprises various redox enzymes, thereby targeting the damage to proteins, DNA, and lipids, ultimately leading to cell apoptosis (Slimen et al. 2014).

Numerous studies have confirmed that heat stress is an important mediator of ROS production, inducing the onset of oxidative stress in the body. O^2−^ is the precursor of most ROS and a key mediator of the oxidative chain reaction (Sies and Jones 2020). Under heat stress, the rate of ferritin release of iron ions is increased in the body, leading to the overproduction of metal ions, which facilitates the binding of electrons to oxygen, resulting in the formation of O^2−^ and H_2_O_2_ (Freeman et al. 1990; Powers et al. 1992). H_2_O_2_ is further reduced to the extremely reactive hydroxyl radical OH via the Fenton reaction (Belhadj et al. 2016). NO has been shown to bind to cytochrome oxidase, thereby stimulating O^2−^ production. Severe hyperthermia stimulates the enhanced local release of NO from the visceral circulation of rats, which leads to increased concentrations of oxygen radicals and metal‐binding proteins in the venous blood (Hall et al. 1985). NADPH oxidase also promotes the production of ROS and the conversion of NADPH to NADP (Fukai and Ushio‐Fukai 2020). High temperatures can activate NADPH oxidase and increase the NADP^+^/NADPH ratio by upregulating NOX1, which is mediated by the ERK signaling pathway (Moon et al. 2010).

In addition to inducing oxidative stress by promoting ROS overproduction, heat stress can exacerbate oxidative stress by disrupting antioxidant defenses in the body. Superoxide dismutase (SOD), an enzyme that removes H_2_O_2_ and lipid hydroperoxides, as well as catalase (CAT) and glutathione peroxidase (GSH‐Px), is the front‐line defense against oxidative stress (Forman and Zhang 2021). Heat stress at 42°C was found to induce a significant decrease in SOD and CAT activities in the brain, liver, and heart of rats, which exacerbated the degree of lipid peroxidation and oxidative stress injury (Yang and Lin 2002). Uric acid is an antioxidant, while allantoin is a ROS marker. After heat exposure at 32°C, SOD and GSH‐Px activities in the chest muscle and thigh muscle of broilers decreased, and plasma allantoin levels increased as the uric acid concentration increased, indicating that excessive heat can stress the antioxidant capacity of the body (Huang et al. 2015). In addition, the burst of ROS under heat stress is closely associated with a remarkable decrease in GSH levels, which might be an early event in heat stress‐induced apoptosis (Sreedhar et al. 2002).

Interestingly, although heat stress induces oxidative stress damage in an organism, which manifests as the overproduction of ROS and damage to the antioxidant system, in the early stages of heat stress, the organism undergoes a series of protective responses to resist oxidative stress damage. For example, heat stress‐activated HSP90 can prevent ROS production and subsequent apoptosis by activating Akt and pyruvate kinase M2 (PKM2) to promote the translocation of B‐cell lymphoma‐2 (Bcl‐2) to the mitochondria and its phosphorylation. In addition, heat stress‐activated HSP90 could inhibit the cAMP response element‐binding protein (CREB)‐ inositol 1,4,5‐trisphosphate (IP3) receptor (IP3R) pathway via the inhibitory effect of Raf‐1‐ERK activation, inhibiting mitochondrial calcium overload, which overcomes mitochondrial permeability transition pore (MPTP) opening and mitochondrial membrane potential (ΔΨm) inhibition (Yao et al. 2023). HSP60 may overcome oxidative stress in cardiomyocyte H9C2 cells (Song et al. 2016). Similar to myocytes, heat stress (43°C)‐treated SW480 cells displayed a time‐dependent increase in intracellular ROS levels, peaking at 6 h, highlighting potential drug targets and strategies for repairing heat stroke‐induced intestinal damage (Song et al. 2016). Intracellular ROS levels increase in C2C12 myoblasts after exposure to heat stress at 43°C for 2 or 4 h (Yu et al. 2019). However, the activities of cytochrome oxidase (CCO) and superoxide dismutase (SOD) did not significantly decrease in the gastric mucosa of rats pretreated with heat shock, and a positive correlation was found between SOD and HSP70 and CCO and HSP60. Therefore, heat shock preconditioning improves burn‐induced acute gastric mucosal lesions and has a protective effect on gastric mucosal mitochondria; the specific mechanism is also related to HSP70 and HSP60 (Zhang et al. 2008). Overall, the organism may trigger a series of protective reactions against oxidative damage after heat stress. Notably, these reactions do not prevent oxidative stress damage; instead, they slow the process of damage.

### 2.3 Heat Stress and Mitochondrial Damage

The mitochondria have diverse and interrelated functions and are the powerhouses of the cell, as they generate most of the energy and aid in the response to cellular stresses, such as autophagy and apoptosis (Annesley and Fisher 2019). The mitochondria maintain an internally negative and externally positive ΔΨm during normal respiratory oxidation. When certain factors impede the electron transfer process in the mitochondrial respiratory chain, the formation of a transmembrane gradient of protons (H^+^) within the matrix is affected, leading to depolarization of ΔΨm (Zorova et al. 2018). According to numerous studies, a decrease in ΔΨm is associated with cellular autophagy, apoptosis, or necrosis. Mitochondrial membrane potential dysfunction, even subtle abnormal changes, may markedly affect intracellular bioactivity and play important regulatory roles in ATP synthesis, ion transport, apoptosis, and antioxidant functions (Zorova et al. 2018). The mitochondria are in a constant state of flux to promote an optimal mitochondrial pool through biogenesis and fusion processes that increase their capacity for ATP synthesis, metabolite sharing, and Ca^2+^ processing; and through mitochondrial fission and mitophagy processes that reestablish metabolic homeostasis and remove excess mitochondria. This balance in mitochondrial regulation can shift toward increased mitochondrial biogenesis and fusion or mitochondrial fission and mitophagy, respectively, when cells are subjected to conditions that disturb metabolic homeostasis (Memme et al. 2021).

At room temperature, the mitochondria are distributed in the cytoplasm as elongated, tubular, and dynamic organelles. During heat exposure, these organelles move to the perinuclear region and form a short, swollen, distended, and compact ring of fused vesicles, and this morphological and structural change is accompanied by perturbation of intermediate filaments (Collier et al. 1993). An increase in temperature from 25°C to 42°C leads to a decrease in ΔΨm, H_2_O_2_ production, and reduced levels of quinone accompanied by uncoupled protein‐mediated proton leakage, which may lead to decreased efficiency of oxidative phosphorylation and ROS generation (Yi et al. 2017; Jarmuszkiewicz et al. 2015). The mitochondria in different tissues and organs are affected by heat stress. Heat stress causes mitochondrial fragmentation and increased expression of the mitochondrial fission protein, dynamin‐related protein 1 (Drp1), in the mouse gastrocnemius muscle; however, the mitochondrial fusion‐related proteins, mitofusin 1 (MFN1), mitofusin 2 (MFN2), and Optic Atrophy 1 (OPA1) are not affected (Yu et al. 2018). The heat stress environment at 42°C leads to reduced mitochondrial content, decreased mitochondrial membrane potential, impaired biogenesis, and stressful elevation of mitophagy levels in C2C12 myoblasts during proliferation and differentiation, resulting in decreased cell viability (Lu et al. 2023). The second mitochondria‐derived activator of caspases (Smac) is a mitochondrial protein that regulates apoptosis. Heat exposure at 42°C induced enhanced expression of HSP70 and HSP90 proteins in cardiomyocytes, which led to the release of Smac from the mitochondria and induced apoptosis. This result indicates that the mitochondrial response to heat stress is closely related to the cellular state, and this injury cannot be rescued by activating the heat shock response (Jiang et al. 2005; Jiang et al. 2003). Early mechanistic studies also revealed the presence of small heat shock proteins (sHSPs) highly expressed in the mitochondria of heat‐stressed rat PC12 cells, which protect the conserved function of mitochondrial electron transport during the stress period (Downs et al. 1999). In the ischemia‐reperfused rat heart, a single heat exposure before ischemia can generate energy by stimulating mitochondrial respiration, controlling ΔΨm and mitochondrial respiration rate, and exerting a protective effect during reperfusion (Pogorzala et al. 2013; Ishikawa et al. 1999). Similarly, in the liver, a single heat exposure reduced the opening of the mitochondrial permeability transition (MPT) pore (He and Lemasters 2003). Therefore, despite the losses associated with heat exposure, a series of protective measures are induced in the organism to prevent further deterioration.

### 2.4 Heat Stress and Autophagy

Autophagy is the primary intracellular degradation mechanism through which cytoplasmic substances are transported and degraded in lysosomes. However, the purpose of autophagy is not simply to eliminate substances but to act as a dynamic recycling system that provides new energy for cell renewal and homeostasis (Mizushima and Komatsu 2011). Autophagy acts as a cellular protective system against cellular dysfunction caused by the excessive accumulation of damaged organelles and denatured proteins during heat stress.

Despite the critical role of autophagy in maintaining cellular homeostasis during heat stress, the temperature at which autophagy is activated has not been clearly defined, especially in humans. The autophagic activity of peripheral blood mononuclear cells (PBMC) isolated from whole blood was found to increase in a temperature‐dependent manner in young people after heating in a water bath set at 37°C, 39°C, and 41°C for 90 min (Mccormick et al. 2020). Similarly, cell models, such as HeLa, HEK293T, and A549 cells, human hepatocellular carcinoma cells, alveolar basal epithelial cells, and human cervical carcinoma cells, exhibited strong activation of autophagy when cultured in thermal environments up to 43°C (Zhao et al. 2009; Han et al. 2013; Dokladny et al. 2013; Nivon et al. 2009). In an animal model, Ganesan et al. found that a single acute heat‐exposure stimulus at 37°C promoted autophagy activation in porcine skeletal muscle based on the enhanced expression of adenosine 5 ‐monophosphate (AMP)‐activated protein kinase‐α (AMPKα) and unc‐51 like kinase 1 (ULK1) phosphorylation; increased expression of the membrane nucleation markers, Beclin‐1 protein and ATG16L‐ATG5‐ATG12 complex protein; and increased conversion of the autophagosome marker, LC3 I to LC3 II. These changes were accompanied by enhanced expression of linear markers of autophagic degradation (Ganesan et al. 2018). Interestingly, although acute thermal stimulation could enhance autophagic activity in porcine skeletal muscle, thermal stimulation at 35°C for 1 or 3 days resulted in dysregulation of its autophagy and elevated expression of the autophagy degradation marker protein, P62, suggesting the failure of autophagosome degradation in skeletal muscle. Brownstein et al. further traced the autophagy upstream signaling pathways, such as AMPK, ULK1, and Beclin1, whose phosphorylation levels did not undergo significant differentiation after 1 or 3 days of heat exposure. Based on their findings, longer heat exposure may induce failure of autophagosome degradation, leading to impaired autophagic flow rather than enhanced autophagy levels (Brownstein et al. 2017). The contradictory results of the above studies may be due to temperature differences in the thermal environments between studies; the mild temperature of 35°C used in the latter study may not cause large protein aggregations that do not require autophagy for clearance. In addition, in a model of senescent rats, the numbers of autophagosomes, autophagic lysosomes, autophagy‐degraded residues, and lipofuscin in the liver of rats exposed to a thermal environment of 41°C–42°C for 0.5 h significantly increased within 48 h after the end of the intervention. In addition to serving as a direct result of thermal stimulation, this significant increase may also be related to the further activation of autophagy by the accumulation of oxides and lipids caused by the decline in metabolic function and heat resistance of aging rats (Oberley et al. 2008).

### 2.5 Heat Stress and Apoptosis

Apoptosis is a unique and important mode of “programmed” cell death and is a homeostatic mechanism for maintaining tissue cell populations during growth, development, and aging. Apoptosis can be induced by different noxious stimuli, such as heat, radiation, hypoxia, and drugs, which may occur as a defense mechanism but may lead to cell death when the dose of these noxious stimuli is too high (D'Arcy 2019). Apoptosis is a coordinated and often energy‐dependent process that involves the activation of a set of enzymes called cysteine asparaginases (caspases) and a complex cascade of events linking the initiating stimulus to cell death (Elmore 2007). Acute heat stress induces apoptosis based on an increase in the Bax/Bcl2 ratio and caspase‐9 and caspase‐3 expression (Yi et al. 2017; Chen, Li, et al. 2014).

Apoptosis is closely related to mitochondrial function, oxidative stress, autophagy, and other pathophysiological processes. The crosstalk among these processes constitutes a profound mechanism by which organisms respond to heat stress. Thermal stimuli induce apoptosis via two mitochondria‐related pathways. Bcl‐2 family proteins can directly modulate the permeability of the outer mitochondrial membrane (OMM) pores (e.g., BCL2‐associated X (Bax)/Bak oligoporous pores and Bax/voltage‐dependent anion channel (VDAC) hybrids) to cytochrome c (Antonsson et al. 2000; Shimizu et al. 2001). Heat stress induces apoptosis by promoting cytochrome c release into the cytoplasm via Bcl‐2 control of mitochondrial Bax translocation (Zhao et al. 2006). In contrast, mitochondrial Ca^2+^ overloading causes the MPTP to remain open, which can lead to nonspecific rupture of the OMM. Thermal stimulation induces Ca^2+^ and ROS overload, leading to cellular matrix swelling and OMM rupture (Zhao et al. 2006). Subsequent hyperpermeabilization of the OMM prompts the release of apoptotic factors, such as cytochrome c and Smac, from the mitochondria into the stroma, where they bind to apoptotic protease‐activating factor 1 (Apaf‐1) to form the Apaf‐1/procaspase‐9 apoptosome, which activates the caspase‐9 signaling cascade to induce apoptosis (Campioni et al. 2005; Kalpage et al. 2019). Oxidative stress also plays an important role in heat stress‐induced apoptosis via the mitochondrial pathway. Heat stress induces apoptosis via the mitochondrial pathway, with mitochondrial p53 translocating in a ROS‐dependent manner, leading to the dysregulation of Ca^2+^ homeostasis and subsequent induction of Bax mitochondrial translocation as an upstream event, triggering the apoptotic process observed in cells exposed to heat stress (Gu et al. 2015).

Autophagy and apoptosis represent a continuum of cellular activation that varies according to the severity of injury (Bloemberg and Quadrilatero 2019). Under physiological conditions, autophagy is maintained at a basal level to sustain normal cellular functions, whereas apoptotic signaling is maintained at a minimum. Autophagy is activated at the onset of intracellular disturbances to remove damaged organelles and proteins. However, when stress exceeds the protective capacity of cellular autophagy for survival, pro‐apoptotic mechanisms are activated, and autophagy is disabled to ensure that the damaged cells are eliminated and damage to other physiological processes is minimized (Chen, Zhou, et al. 2014; Kovacs et al. 2012). Autophagy remains the main stress response pathway in human PBMCs heated for 90 min at 41°C (Mccormick et al. 2020). Heat treatment is cytotoxic when the cell temperature exceeds 42.5°C (van der Zee 2002), and stronger apoptotic signaling is demonstrated in human HeLa cells after 3 h of incubation at 43°C in vitro (Bettaieb and Averill‐Bates 2015). Interestingly, another study found increased levels of markers of apoptotic signaling after 2 h of heat stimulation; however, the levels returned to baseline thereafter, while caspase 3 activity remained elevated by 2–3‐fold throughout the heat stimulation period. In contrast, markers of autophagy increased only after 6 h, suggesting that apoptotic signaling may precede an increase in autophagy during acute heat stress; however, mitochondrial autophagy is triggered (Ganesan et al. 2018). The over‐activation of autophagy under heat stress is not a protective response. Lu et al. found that heat stress enriched intracellular ROS in myofibroblasts and induced strong expression of the Bcl‐2 protein, which led to the activation of excessive autophagy and accelerated apoptosis (Lu et al. 2023). The protective mechanism generated by the body under heat exposure is associated with the inhibition of Bax activation via the overexpression of HSPs, such as HSP70 (Stankiewicz et al. 2005). Overall, the initiation of apoptosis may be due to the activation of inflammation, autophagy, and oxidative stress signaling owing to heat stress.

## 3 Heat Stress‐Induced Multi‐Organ Damage: Physiological and Pathological Mechanisms

As global temperatures rise, heat waves become more frequent, intense, and longer‐lasting, putting a greater proportion of the population at deadly risk from extreme heat (Mora et al. 2017). Heat exposure increases the risk of heat syncope, heat exhaustion, and even heat stroke, leading to systemic inflammation, cardiovascular dysfunction, altered cardiovascular function, and other multi‐organ system damage, ultimately leading to death (Bouchama et al. 2022; Armstrong et al. 2007). In the previous section, we summarized some of the pathological changes in organismal physiological processes in response to heat stress, which often leads to further multi‐organ damage such as central nervous system damage, intestinal dysfunction, hepatic damage, cardiac dysfunction, and skeletal muscle dysfunction, but the specific mechanisms through which heat stress induces these pathological changes are not known. In the next section, we will focus on the pathological mechanisms involved in heat stress‐mediated multi‐organ damage.

### 3.1 Central Nervous System Damage

**FIGURE 3:**
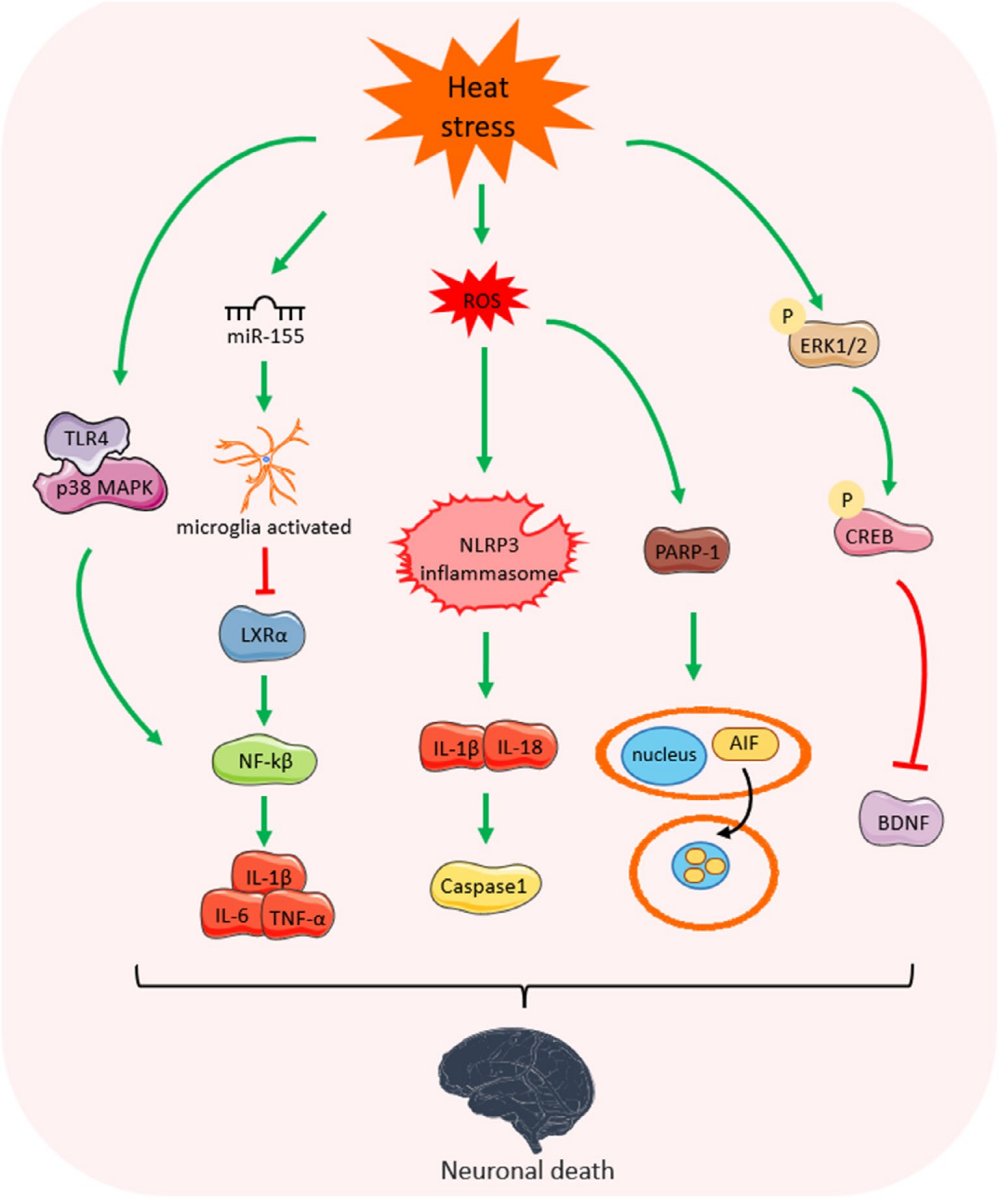
Molecular mechanisms of heat stress‐induced CNS injury. Heat stress can activate NF‐kβ signaling through TLR4/p38 MAPK and miR‐155/LxRα pathways, which promote the release of inflammatory factors IL‐1β, IL‐6, and TNF‐α. Heat stress‐induced oxidative stress also contributes to neuroinflammation and causes neuronal apoptosis. In addition, heat stress‐induced oxidative stress activates PARP‐1 signaling and induces AIF translocation from the cytoplasm to the nucleus, leading to PARP‐1‐dependent cell death in neurons. During severe heat stress, phosphorylation activation of ERK1/2 promotes CREB phosphorylation, which inhibits BDNF expression.

The central nervous system is most sensitive to heat stress, and high temperatures can directly damage the central nervous system, inducing several pathophysiological changes in brain cells, including lipid peroxidation of membranes, reduced cerebral perfusion, altered fluidity of brain cell membranes, and reduced activity of membrane receptors and enzymes, which leads to metabolic disorders, cerebral edema, and even the development of degeneration and necrosis of brain cells (Li et al. 2015; Howells et al. 2013; Mcgeehin and Mirabelli 2001). In addition, the main pathological basis of brain injury under heat stress may also be the rapid death of central nerve cells due to ischemia and hypoxia in brain tissue. Among them, the damage to cortical pyramidal cells and small Purkinje cells, as well as to hyperthermia‐sensitive neurons, is more severe (Yeo 2004) (Figure 3).

In heat stress‐mediated brain injury, the central neuroinflammatory response plays an extremely critical role. Sajan Mahajan's case report showed that cranial magnetic resonance in patients with pyrexia revealed abnormal high signals in the cerebral white matter bilaterally in the cortex and sub‐hippocampus, which suggests that a significant inflammatory response occurs in the brain tissue. The elevation of inflammatory factors IL‐1, IL‐6, etc. in brain tissue after pyrexia correlates significantly with neurological damage, angioedema, and neuronal death (Leon et al. 2006). The cortex and hippocampus are the most common sites of injury in the central system, and the onset and development of inflammatory responses in the central nervous system are closely related to microglia. In animal models of heat stress, activated microglia were found in the cerebral cortex, hippocampus, and pituitary gland. Interestingly, the expression of microglia activation markers was consistent with the expression of cytokines and chemokines in heat‐stressed mice. This suggests that microglia may play a critical immunomodulatory role in the central nervous system during heat stress (Biedenkapp and Leon 2013). When the central nervous system is subjected to external pathological stimuli or injury, microglia are rapidly activated and undergo functional and morphological changes. Activated microglia mainly show two different activation phenotypes: M1‐type activation and M2‐type activation; M1‐type activated microglia mainly secrete pro‐inflammatory factors such as TNF‐α, IL‐1β, IL‐6, nitric oxide, etc., which aggravate CNS injury; M2‐type activated microglia mainly secrete inhibitory factors such as IL‐10, IL‐13, IL‐4, transforming growth factor β, and various neurotrophic factors, etc., which mainly play the role of immunomodulation, promote nerve repair and regeneration, and play the role of neuroprotection. M2‐type activated microglia mainly secrete anti‐inflammatory factors IL‐10, IL‐13, IL‐4, transforming growth factor β, and various neurotrophic factors, etc., which mainly play the roles of immunomodulation and promotion of nerve repair and regeneration (Nakagawa and Chiba 2014; Loane and Kumar 2016; Chio et al. 2015). Heat stress‐induced microglia activation in mouse brain tissue and the pattern of activation was mainly M1‐type polarization 1–3 h after heat stress, and M2‐type polarization 24 h after pyrexia, which indicated that the transformation of microglia from M1‐type to M2‐type after heat stress might be the main feature of the development of CNS inflammation in pyrexia (Zhu et al. 2023). Further mechanistic studies revealed that micro RNAs may be involved in microglia activation under heat stress. miR‐155 was the first miRNA reported to be directly associated with microglia activation, and inhibition of miR‐155 ameliorated ischaemic stroke in mice by reducing the production of pro‐inflammatory mediators (Wen et al. 2015). Heat stress promotes the expression of miR‐155 in microglia and is accompanied by an increase in the release of IL‐1β, IL‐6, and TNF‐α. This is due to the ability of miR‐155 to target and inhibit the expression of LXRα, which promotes the activation of the NF‐κB pathway to increase the expression of inflammatory factors, ultimately leading to neurotoxicity and inflammatory damage in the brain (Li, Wang, et al. 2019). In addition, NF‐κB signaling activated by the TLR4/p38 MAPK pathway is also a mechanism of heat stress‐induced neuroinflammation (Huang et al. 2022). Furthermore, heat stress‐induced excessive ROS production during the attack on microglia, followed by activation of the NLRP3 inflammasome in mouse and BV2 microglia. With the activation of the NLRP3 inflammasome, the expression levels of IL‐1β, IL‐18, and caspase‐1 were elevated. In NLRP3 knockout mice, heat stress‐mediated expression of IL‐1β, IL‐18, and caspase‐1 was significantly decreased. This suggests that heat stress causes neuroinflammation by activating the NLRP3 inflammasome in microglia through the generation of excessive ROS (Du et al. 2024).

Oxidative stress‐induced DNA damage is also a cause of heat stress‐induced brain damage. Poly (ADP‐ribose) (PAR) polymerase‐1 (PARP‐1), a key factor in DNA damage repair and cell survival, mediates parthanatos in response to excessive accumulation of ROS (Yu et al. 2002). Recent studies have found that heat stress activates PARP‐1/AIF signaling in vivo and in vitro, inducing AIF translocation from the cytoplasm to the nucleus and leading to PARP‐1‐dependent cell death in neurons (Wang et al. 2023). During severe heat stress, high levels of oxidative stress in rat hippocampal tissues lead to neuronal damage and apoptosis in the CA3 region of the hippocampus, ultimately resulting in spatial memory deficits. The mechanism may be that heat stress induces the activation of the phosphorylated extracellular signal‐regulated kinase pERK 1/2, which leads to further activation of the phosphorylated cAMP‐responsive element‐binding protein (pCREB), preventing it from interacting with the coactivator CREB‐binding protein (CBP) and leading to a further decrease in BDNF expression (Chauhan et al. 2021).

### 3.2 Intestinal Dysfunction

**FIGURE 4:**
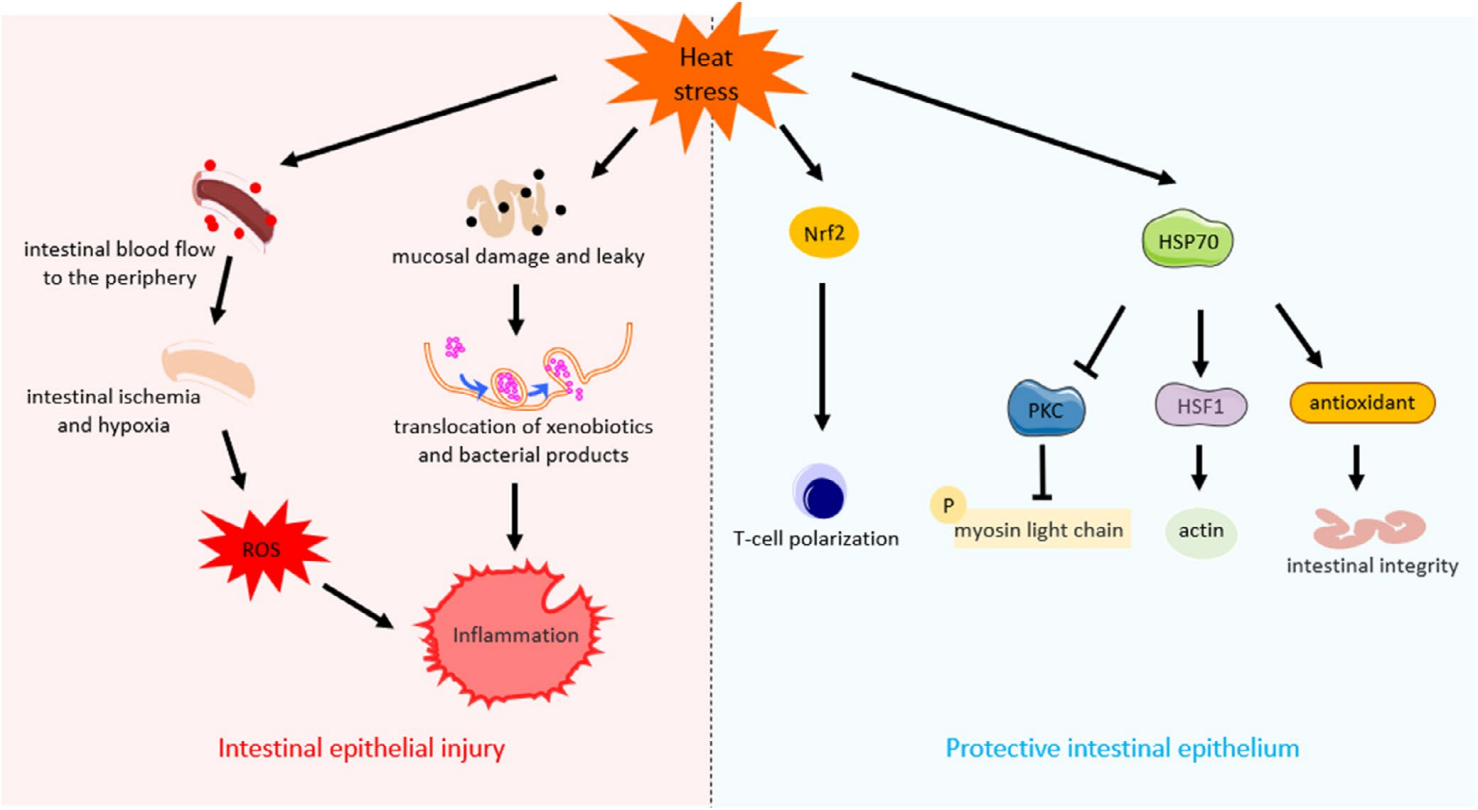
Molecular mechanisms of heat stress‐induced intestinal dysfunction. Ischemia and hypoxia in the intestines under heat stress lead to disturbances in the balance between oxidative and antioxidant defenses, causing oxidative stress and an inflammatory response. Heat stress‐induced mucosal damage and leaky gut can lead to translocation of xenobiotics and bacterial products, which cause inflammation and exacerbate intestinal dysfunction. In addition, there are certain defense mechanisms in the intestinal system under heat stress: Nrf2 Can regulate intestinal immune function and inhibit inflammation by affecting T‐cell polarization, and HSP70 can prevent PKC activation and reduce myosin light chain protein phosphorylation. At the same time, HSP70 can promote the expression of HSF1, increase the expression of actin fibers in intestinal epithelial cells, and resist intestinal epithelial damage caused by oxidative stress.

The intestinal epithelium is the physical and biochemical barrier between pathogenic microbial communities and the intestinal mucosal immune system. Various pathological or physical stressors, including heat stress, can lead to intestinal epithelial barrier dysfunction, resulting in local or systemic inflammatory responses (Lambert 2009; Peterson and Artis 2014). Severe intestinal epithelial damage is considered a major factor in heat stress mortality (Bouchama and Knochel 2002). During heat stress, the body's thermoregulatory mechanisms prompt intestinal blood flow to circulate peripherally to promote heat dissipation, which leads to ischemia and hypoxia in the gut. Heat stress‐induced hypoxic conditions in the gut lead to a disturbed balance between oxidative and antioxidant defenses, resulting in epithelial damage and inflammatory responses (Lambert et al. 2002) (Figure 4). Intestinal hypoxia can also induce local inflammation through barrier‐independent pathways, including cellular acidification by glycolysis, activation of autophagy, and innate protective immune responses triggered by hypoxia‐inducible factor‐1α (HIF‐1α) (Yamoto et al. 2019; Shah 2016). In addition, heat stress‐induced mucosal damage and leaky gut can lead to the translocation of xenobiotics and bacterial products, which can cause inflammation and exacerbate intestinal dysfunction (Zuhl, Schneider, et al. 2014). Heat stress‐induced intestinal barrier dysfunction is associated with the expression of tight junction and adhesion junction proteins as well as altered cellular localization, which requires a functional junction complex to seal the paracellular space between neighboring cells, thereby preventing luminal antigen and microbial penetration (Varasteh et al. 2015; Zuhl, Lanphere, et al. 2014). Further studies revealed that some protective mechanisms still exist in the intestinal epithelium under heat stress, and the activation of HSPs has been reported to have a certain mitigating effect on intestinal epithelial damage under heat stress. In particular, the expression of HSP70 is associated with the stabilization of the actin cytoskeleton of intestinal cells, thus preventing their aggregation under stress conditions (Dokladny et al. 2016). Heat stress‐mediated activation of HSP70 induces HSF1 binding to the occludin promoter region, which promotes HSF1 expression and improves the involvement of tight junction‐associated proteins (occludin) in the junctional complex, ultimately increasing the expression of actin in the intestinal epithelium, and to a certain extent, resisting heat stress‐induced intestinal epithelial damage (Fukai and Ushio‐Fukai 2020). The addition of exogenous HSP70 to intestinal epithelial cell line Caco‐2 cultures prevents heat stress‐induced alterations in cell permeability (Varasteh et al. 2018). In addition, one of the mechanisms by which the antioxidant α‐lipoic acid and the amino acid arginine maintain intestinal integrity may be related to enhanced HSP70 expression (Varasteh et al. 2018). Another possible mechanism by which HSP70 attenuates intestinal epithelial barrier dysfunction under heat stress conditions is by preventing the activation of protein kinase C(PKC), thereby reducing myosin light chain protein phosphorylation in the actin cytoskeleton (Zuhl, Schneider, et al. 2014; Yang et al. 2007). In addition to the neuroprotective effects of HSP70, nuclear factor‐erythroid 2‐related factor 2(Nrf2) has been shown to modulate intestinal immune function by influencing T cell polarization. Activation of Nrf2 suppresses Th1 cytokine (IFNγ) and IL‐2 secretion during an early stage of heat stress and then promotes CD4 Th2 differentiation (Weigand et al. 2007; Kew et al. 1978).

### 3.3 Liver Injury

**FIGURE 5:**
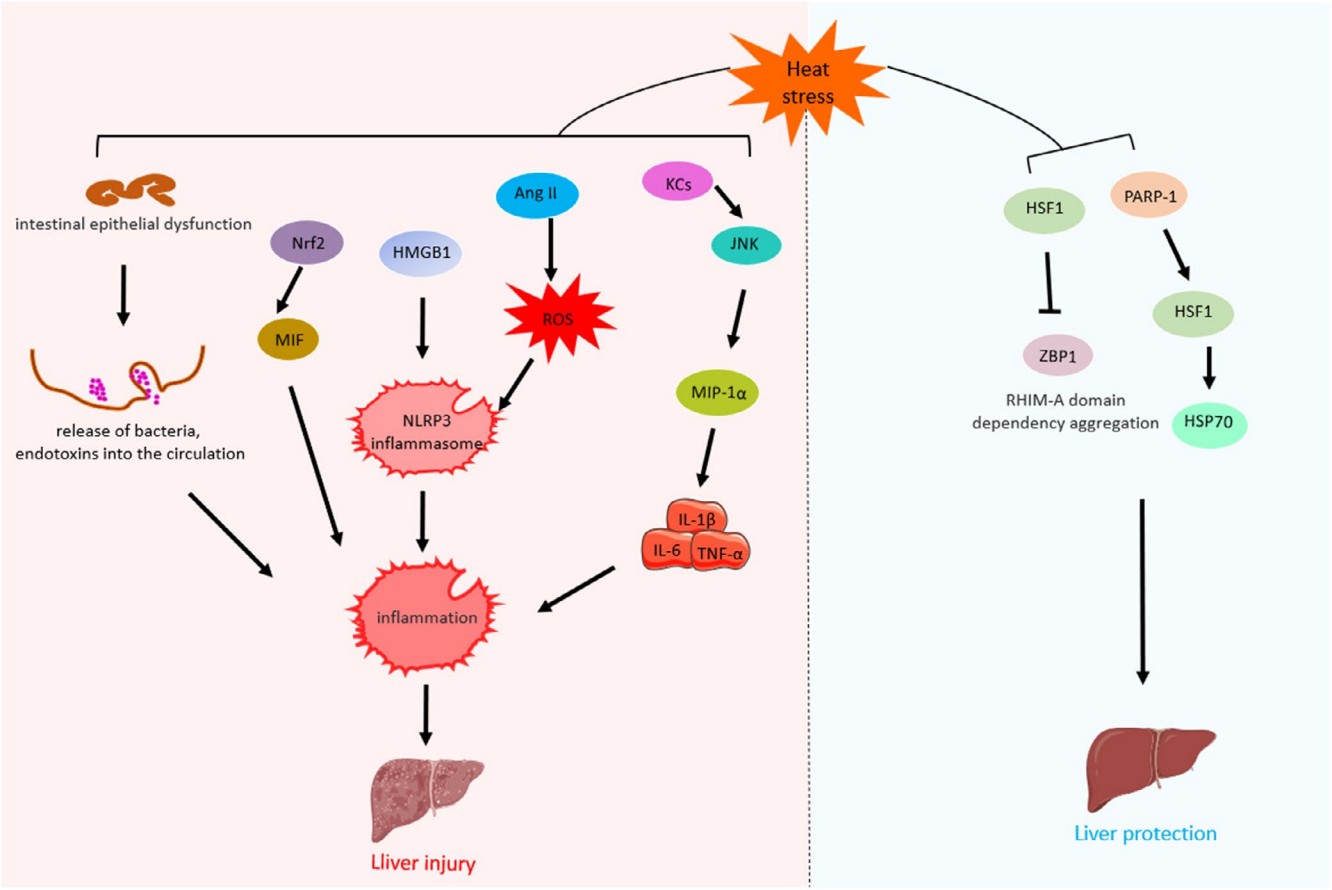
Molecular mechanisms of heat stress‐induced liver injury. Heat stress‐induced intestinal epithelial dysfunction results in the translocation of bacteria and endotoxins from the gut to the circulation, leading to an excessive inflammatory response in vivo and contributing to hepatocyte degeneration. Pathways involved in the heat stress‐induced hepatic inflammatory response include Nrf2‐MIF, HMGB1‐NLRP3 inflammasome, Ang II‐ROS, and JNK‐MIP‐1 pathway. Some protective mechanisms still exist in heat stress‐induced liver injury. Inhibition of PARP‐1 expression under heat stress promotes HSF1 expression, which in turn induces HSP70 expression and reduces liver injury.

Acute liver injury and even more severe acute liver failure are direct causes of death in heat stress patients (Weigand et al. 2007) (Figure 5). Blood from the gastrointestinal tract must flow into the portal vein in order to enter the liver, and heat stress‐induced intestinal epithelial dysfunction results in the translocation of bacteria and endotoxins from the gut into the circulation, leading to an excessive inflammatory response in the body and contributing to the massive degenerative changes in hepatocytes (Kew et al. 1978; Garcin et al. 2008). For an in‐depth evaluation of liver function during severe heat stress, Gupta et al. analyzed the hepatic transcriptome and proteome of male Sprague Dawley rats subjected to heat stress at 45°C using microarray and two‐dimensional gel electrophoresis, respectively, and found significant alterations in redox state, inflammation, mitochondrial dysfunction, and proteostasis‐related pathways. Nrf2‐mediated oxidative stress and macrophage migration inhibitory factor (MIF)—regulated inflammatory pathways were up‐regulated in heavily heat‐stressed livers (Gupta et al. 2022). In addition, the NOD‐like receptor family pyrin domain containing 3 (NLRP3) is an intracellular pattern recognition receptor that participates in cellular cell death by assembling into inflammatory vesicles (Wree et al. 2014). NLRP3‐dependent pyroptosis is a key cause of heat stress‐induced aberrant hepatocyte death. Heat stress‐induced liver injury is often accompanied by activated inflammatory vesicles, which can effectively induce IL‐1β activation and hepatocyte cell pyroptosis, leading to severe liver injury (Geng et al. 2015). HMGB1, a typical damage‐associated molecular pattern molecule, can mediate heat stress‐induced activation of the NLRP3 inflammasome via Toll‐like receptor 4 and late glycosylation end product receptor signaling, leading to hepatocyte scorched death and severe liver injury. After 4 h of exposure to 38°C, mice showed significantly higher HMGB1 concentrations accompanied by increased concentrations of aspartate aminotransferase and alanine aminotransferase, whereas inhibition of HMGB1 expression significantly reduced the expression of cellular inflammatory factors and reduced the area of hepatocyte necrosis (Kawasaki et al. 2014). In addition, excess ROS produced during heat stress has been shown to be a key stimulus for the NLRP3 inflammasome and a potential target for the negative regulation of cellular pyroptosis (Liu et al. 2017; Tschopp and Schroder 2010). Angiotensin II (Ang II) and Ang‐(1–7) are potential markers of inflammatory diseases; their levels change during heat stress and are strongly associated with ROS overproduction (Pan et al. 2014). In heat stress‐induced liver injury, Ang II expression is increased while Ang‐(1–7) levels are decreased. Increased Ang II levels further enhanced the excessive focal death response of hepatocytes induced by the ROS‐NLRP3 pathway (Zhang et al. 2019). In contrast, AVE 0991, an Ang‐(1–7) analog, significantly inhibited ROS production and reduced protein levels of NLRP3, IL‐1β, NOX4, and caspase‐1 in hepatocytes under heat stress, thereby attenuating hepatocyte degeneration (Zhang et al. 2019).

In addition to the production of inflammatory responses in hepatocytes during heat stress, hepatic resident macrophages/Kupffer cells (KCs) are also a major source of hepatic inflammatory factors, and the mechanism may be that phagocytosis of KCs is impaired, leading to an increase in the concentration of endotoxin that clears intestinal sources (Kolios et al. 2006; Fukuda et al. 2006). TNF‐α, IL‐1β, and IL‐6 secreted by KCs were significantly increased in heat stress, and inhibition of the secretion of KCs attraction‐related factors could effectively alleviate heat stress‐induced liver injury (Chen et al. 2013). Macrophage inflammatory protein‐1α (MIP‐1α) is an important chemokine in the inflammatory response, and KCs are the main source of MIP‐1α in the liver (Liu et al. 2015). In heat stress‐induced liver injury, MIP‐1α expression was significantly increased in KCs, which further promoted the downstream secretion of TNF‐α, IL‐β, and IL‐6, a process over which the activation of JNK signaling was closely related (Gonda et al. 2012; Shen et al. 2005).

Z‐DNA‐binding protein 1 (ZBP1)‐mediated programmed cell death is another mechanism leading to hepatocyte degeneration under heat stress. Activation of ZBP1 normally requires binding of the Zα domain of Z‐nucleic acids and activation of the RHIM domain of RIPK3. However, the Zα domain is dispensable for ZBP1 activation under heat stress, and RHIM‐A domain‐dependent aggregation of ZBP1 is a direct cause of cell death in hepatocytes under heat stress. Endogenous Z‐nucleic acids may enhance heat stress‐induced ZBP1 activation because deletion or mutation of the Zα or Zα2 domains slightly inhibits heat stress‐induced hepatocyte death. In addition, heat stress enhances activation and occupancy of the heat shock transcription factor 1 (HSF1) binding site in the ZBP1 promoter, whereas deletion of HSF1 inhibits heat stress‐induced increase in ZBP1 expression and hepatocyte death (Yuan et al. 2022).

In addition, certain protective mechanisms still exist in heat stress‐induced liver injury, the most important of which is the increased expression of HSP. In both heat‐stressed patients and heat‐stressed animal models, HSP levels were elevated to varying degrees to protect the cells from heat injury to a certain extent (Wang et al. 2001). Increased expression of HSP70 was found to be effective in preventing liver injury, and pretreatment with 17‐dimethylaminoethyl‐17‐demethoxy‐gelatinomycin up‐regulated the expression of HSP70 in the liver, thereby significantly alleviating heat stress‐induced liver dysfunction in rats (Tsai et al. 2016). Inhibition of poly(ADP‐ribose) polymerase‐1 (PARP‐1), a highly conserved nuclear zinc finger DNA‐binding protein, reduces various forms of liver injury (Mukhopadhyay et al. 2017). Expression of HSP70 and HSP27 was significantly elevated in the livers of PARP knockout mice in heat stress‐induced liver injury (Mota et al. 2008). The up‐regulation of HSP proteins and the inhibition of PARP show concordance in heat stress‐induced liver injury, which on the one hand may be due to the fact that PARP‐1, as part of the histone variant mH2A1.1 complex, is associated with the HSP70 promoter, which inactivates HSP70 expression (Ouararhni et al. 2006), and on the other hand the possibility is that PARP‐1 disrupts the DNA binding of HSF1 and the promoter of the HSP gene (Fossati et al. 2006).

### 3.4 Cardiovascular System Damage

**FIGURE 6:**
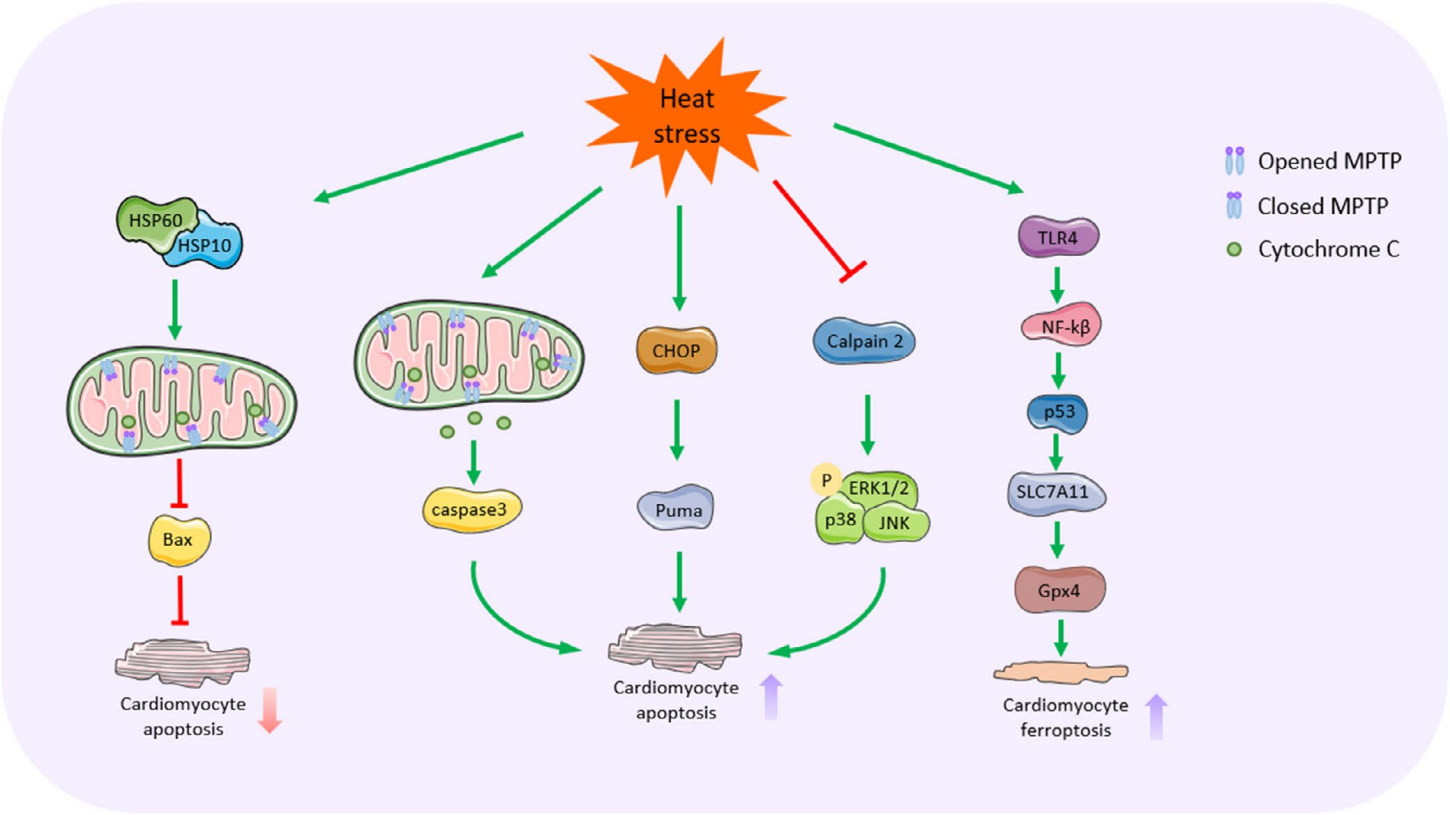
Molecular mechanisms of heat stress‐induced injury to the cardiovascular system. Heat stress is able to induce cardiomyocyte apoptosis through three pathways: (1) Heat stress induces the opening of mitochondrial MPTP, releases cytochrome c into the cytoplasm, and activates the caspase‐3 signaling cascade, which leads to apoptosis of cardiomyocytes. (2) Heat stress activates CHOP/Puma signaling, which induces excessive ER responses and promotes cardiomyocyte apoptosis. (3) Heat stress inhibits the expression of calpain 2 and promotes the phosphorylation of p38, ERK1/2, and JNK, which induces cardiomyocyte apoptosis. Iron death induced by the TLR4/NF‐kβ/p53/SLC7A11/Gpx4x pathway is another novel mechanism of cardiomyocyte death. In addition, certain protective mechanisms still exist in the myocardium under heat stress. HSP60 binds to HSP10 to form a mitochondrial complex that regulates MPTP opening, inhibits mitochondrial dysfunction and Bax expression, and thus alleviates myocardial apoptosis to a certain extent.

The cardiovascular system is a major participant in the systemic heat dissipation response and organ perfusion during heat stress, with cardiovascular dysfunction occurring in 43.4%–65.2% of multi‐organ injuries caused by heat stress (Liu et al. 2019) (Figure 6).

#### 3.4.1 Heart Dysfunction

Cardiac function is most likely to be impaired during heat stress, manifesting as arrhythmias, heart failure, focal myocardial necrosis, etc. (Desai et al. 2023). Heat stress‐induced cardiac dysfunction is not only due to a decrease in cardiac output per beat as a result of increased heart rate and decreased circulating blood volume but also due to a decrease in the contractile capacity of cardiomyocytes in the presence of high temperatures. Heat stress causes a variety of pathological effects on the cardiovascular system, such as mitochondrial dysfunction, oxidative stress, inflammation, and apoptosis (Chen et al. 2017; Lin et al. 2017). Mitochondria are critical for maintaining normal myocardial function, and in heat stress‐induced injury to the myocardium, mitochondrial functions such as respiratory control rate and oxidative phosphorylation efficiency decrease progressively with increasing body core temperature. Energy production from mitochondrial oxidative metabolism is inhibited during heat stress, whereas heat exposure induces an increase in cardiac function that requires a greater supply of energy, resulting in a significant decrease in ATP levels in cardiomyocytes. It is due to the uncoupling of oxidative phosphorylation that heat stress induces myocardial structural dysfunction as well as increased body heat load, ultimately leading to cardiac fatigue. In addition, heat stress directly induces the opening of mitochondrial MPTP, which leads to changes in mitochondrial MPT. Changes in mitochondrial MPT lead to the release of cytochrome c from mitochondria into the cytoplasm of cardiomyocytes, which activates the caspase‐3 signaling cascade, leading to cardiomyocyte apoptosis (Qian et al. 2004). Further studies revealed that the apoptotic cascade was also mediated by activation of the PERK/eIF2α/CHOP unfolding protein response, which in turn up‐regulated the protein expression of Puma, a downstream pro‐apoptotic gene associated with excessive ER stress. Apoptosis in response to heat stress was significantly reduced in CHOP siRNA‐transfected cardiomyocytes, suggesting that CHOP/Puma activation is critical for heat stress‐induced apoptosis in cardiomyocytes (Yang et al. 2014). In addition, calpain‐2 plays a key role in heat stress‐mediated myocardial apoptosis. Heat stress decreased calpain activity in primary mouse neonatal cardiomyocytes (MNC), accompanied by downregulation of calpain‐2 expression and increased phosphorylation of p38, extracellular signal‐regulated protein kinase (ERK1/2), and c‐Jun N‐terminal kinase (JNK), and blockade of p38 alone prevented heat stress‐induced apoptosis in MNC cells. Apoptosis induced by heat stress. In cardiac‐specific calpain‐2 overexpressing transgenic mice, p38 phosphorylation and apoptosis were significantly reduced in the myocardium after heat stress intervention, and M‐mode echocardiography also demonstrated that calpain‐2 overexpression significantly ameliorated heat stress‐induced reductions in ventricular end‐diastolic volume and cardiac output (Liu et al. 2019).

Furthermore, TLR4‐mediated iron death is a novel mechanism of heat stress‐induced myocardial injury. TLR4 is a pattern recognition receptor that regulates various modes of cell death in myocardial tissues, such as apoptosis, autophagic death, and iron death (Chen et al. 2019). TLR4 expression is significantly elevated in cardiomyocytes under heat stress, and one of its downstream molecules, p53, acts on SLC7A11 and inhibits the expression of SLC7A11 and GPX4, thereby inducing iron death (Zhu et al. 2011). In addition, TLR4 is able to activate the NF‐κB pathway to upregulate p53 expression, which leads to heat stress‐induced iron death and exacerbates the cardiomyocyte inflammatory response and injury (Chen et al. 2023).

A protective mechanism also exists during heat stress‐induced cardiac injury, where small oligomers formed by Hsp27 maintain cytoskeletal integrity (Huot et al. 1996), which is necessary to maintain normal muscle function. αB‐crystallin accounts for 3% of total cardiac protein, and overexpression of αB‐crystallin in cultured cardiomyocytes protects them from ischemia‐induced cell death and stabilizes microtubules (Garrido et al. 2012). The distribution densities of Hsp27 and αB‐crystallin in rat cardiomyocytes were reported to increase with the duration of heat stress and were different from those in control cells even after a short exposure to heat stress. Although Hsp27 and αB‐crystallin were distributed in the nucleus and cytoplasm of control and heat‐stressed cardiomyocytes, stronger signals were found in the cytoplasm of heat‐stressed cells, especially after 240 min of exposure, which may be related to their role in heat‐stress‐induced denatured protein replication (Tang, Buriro, et al. 2013). In addition, higher densities of HSP10 and HSP60 positive signals were detected in the heat‐stressed group compared with the control rat myocardium, and heat‐stressed cells exhibited a punctate distribution in the mitochondria. These molecules can be transported into mitochondria under heat stress conditions, and Hsp60 can bind to Hsp10 to form a mitochondrial complex, thus maintaining mitochondrial integrity and ATP production and slowing down mitochondrial dysfunction in heat‐injured myocardium to a certain extent (Cheng et al. 2016; Lau et al. 1997). Further studies revealed that Hsp60 regulates MPTP through mitochondrial outer membrane permeabilization in response to heat stress, thereby mitigating apoptosis, whereas knockdown of Hsp60 in cardiomyocytes resulted in mitochondrial dysfunction, exacerbated ROS production, and induced the expression of Bax, which intensified apoptosis in cardiomyocytes (Song et al. 2016).

#### 3.4.2 Vascular Dysfunction

Vascular endothelial cells are important target organs and effectors in heat stress‐induced cardiovascular injury. Vascular endothelial cells during severe heat stress are continuously exposed to a range of shear stresses generated by changes in blood flow, which may have a significant impact on the function and structure of the vascular endothelium. Hyperthermia stress may also directly induce vascular endothelial cell injury and death through apoptosis, extensive hemorrhage, thrombosis, and leukocyte transmigration (Roberts et al. 2008). Heat stress inhibits platelet release from the bone marrow due to the sensitivity of megakaryocytes to heat exposure. The clinical manifestation of heat stress‐induced coagulation activation and fibrin formation is coagulation dysfunction (DIC) (Bouchama and Knochel 2002). In a study of 18 patients with severe heat stroke, whole blood tissue factor, prothrombin‐antithrombin complex, and soluble thrombomodulin levels were dramatically elevated, whereas levels of the major physiological anticoagulants, including platelets, antithrombin, protein C, and protein S, were significantly reduced (Huisse et al. 2008). Extensive hemorrhage and thrombosis, leukocyte migration, and microvascular endothelial damage with increased staining for endothelial vascular hemophilic factor, tissue factor, and endothelial leukocyte‐platelet interactions were observed in heatstroke baboons (Dokladny et al. 2008). In addition, levels of D‐dimer and soluble thrombomodulin were elevated in the early and middle stages of pyrexia, whereas D‐dimer levels were reduced during the period of most severe organ damage, suggesting potential fibrinolysis‐resistant thrombosis (Proctor et al. 2020). Thrombomodulin is a transmembrane proteoglycan located on the surface of vascular endothelial cells with anticoagulant properties and a soluble form of thrombomodulin that is released from membrane‐bound thrombomodulin, which can be proteolytically released into the circulation and is considered to be a marker of vascular endothelial cell injury (Martin et al. 2013). Exogenous administration of recombinant soluble thrombomodulin ameliorates DIC and reduces mortality in pyrexia group mice (Kawasaki et al. 2014). The above results suggest that heat stress or even heatstroke‐induced DIC has potential staging variability and that coagulation markers can be used as indicators to monitor the severity of heat stress and the course of treatment, but the exact mechanism of action is not clear.

### 3.5 Skeletal Muscle Damage

**FIGURE 7:**
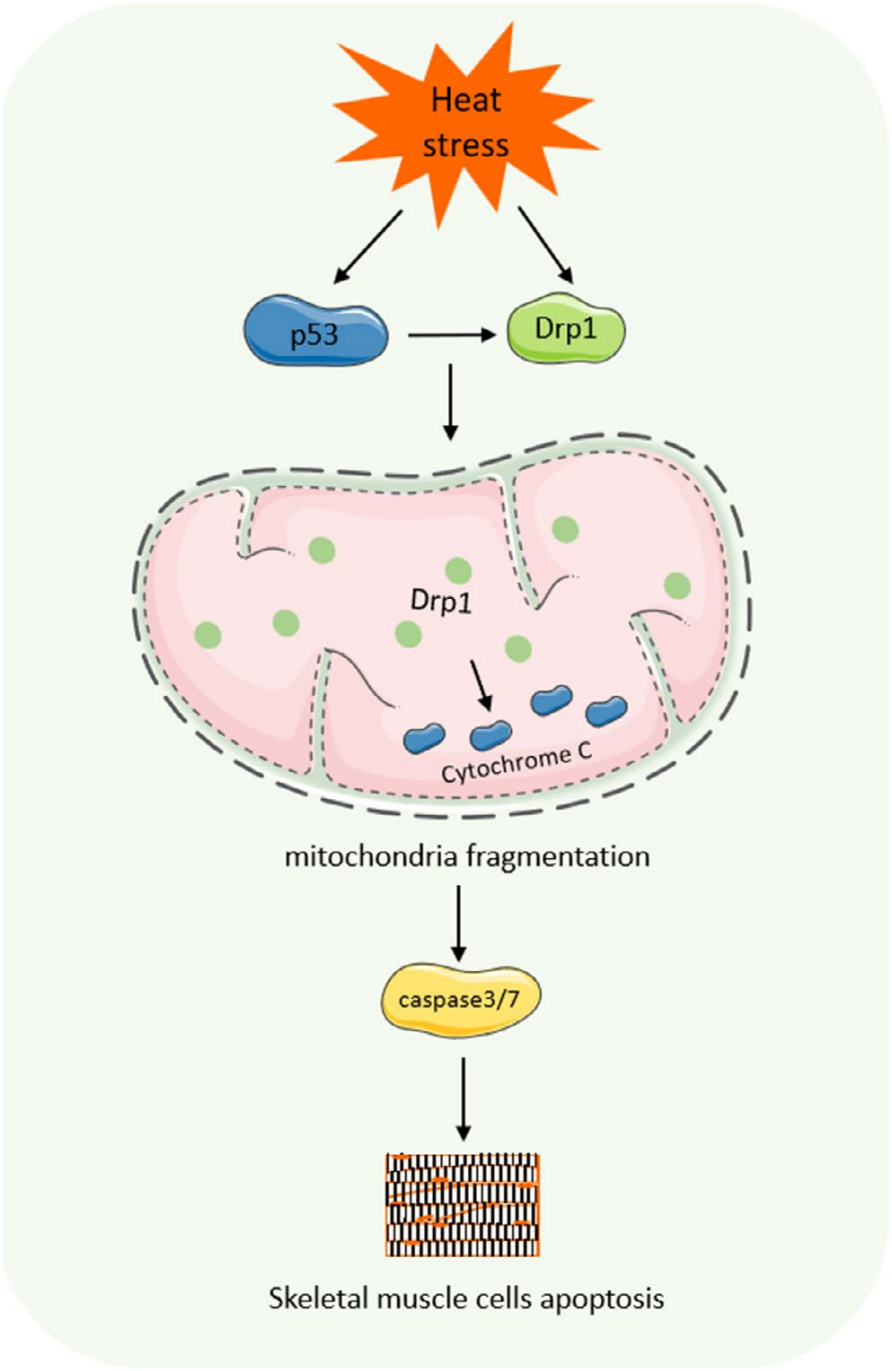
Molecular mechanisms of heat stress‐induced skeletal muscle injury. Heat stress activates the p53 protein, induces translocation of Drp1 from the cytoplasm to the mitochondria, promotes the release of cytochrome c, leads to excessive mitochondrial fragmentation, and contributes to the activation of caspase 3/7, thereby inducing apoptosis in skeletal muscle cells.

Skeletal muscle accounts for about 40% of total body mass and is highly susceptible to heat injury (Figure 7). Previous studies have shown that heat stress‐induced skeletal muscle injury is closely related to mitochondrial dysfunction and overproduction of ROS. Heat‐exposed mice exhibit higher levels of ROS, cleaved caspases, fragmented DNA, and Drp1 protein expression in gastrocnemius muscle (Chen and Yu 2021). Heat stress induces translocation of mitochondrial fission GTPase dynamin‐related protein 1 (Drp1) from cytoplasmic lysosomes to mitochondria, leading to excessive mitochondrial fragmentation and loss of mitochondrial membrane potential in C2C12 myoblasts, which contributes to caspase 3/7 activation, cytochrome c release, and loss of membrane integrity, whereas mitochondrial fission inhibitor 1 or Drp1 gene silencers are not effective in heat‐exposed mitochondrial fragmentation, which was reduced and cell viability increased during exposure to heat stress, suggesting that Drp1‐dependent mitochondrial fission may modulate the susceptibility of muscle cells to heat stress‐induced apoptosis (Yu et al. 2016). However, the mechanism by which acute heat stress leads to upregulation of Drp1 in mouse skeletal muscle may be that heat stress activates the p53 protein, leading to increased levels of Drp1 in tissues. In vitro experiments have also shown that incubation at high temperatures for 2 h leads to p53 activation and mitochondrial translocation, which in turn leads to transcriptional activation and increased Drp1 expression (Gu et al. 2014; Li et al. 2010). Mitochondria are instant sites of free radical production and targets of free radical attack, which tightly links oxidative stress to mitochondrial dysfunction (Lee et al. 2012). Oxidative damage observed during heat stress increases the likelihood of mitochondrial dysfunction, leading to increased free radicals and heat production (Kiffin et al. 2006). Heat stress‐induced free radical damage and inflammation occur in oxidative but not in glycolytic muscles (Montilla et al. 2014), further implying that mitochondria are central to heat stress‐mediated pathology.

## 4 Treatment Strategies for Heat Injury

**FIGURE 8:**
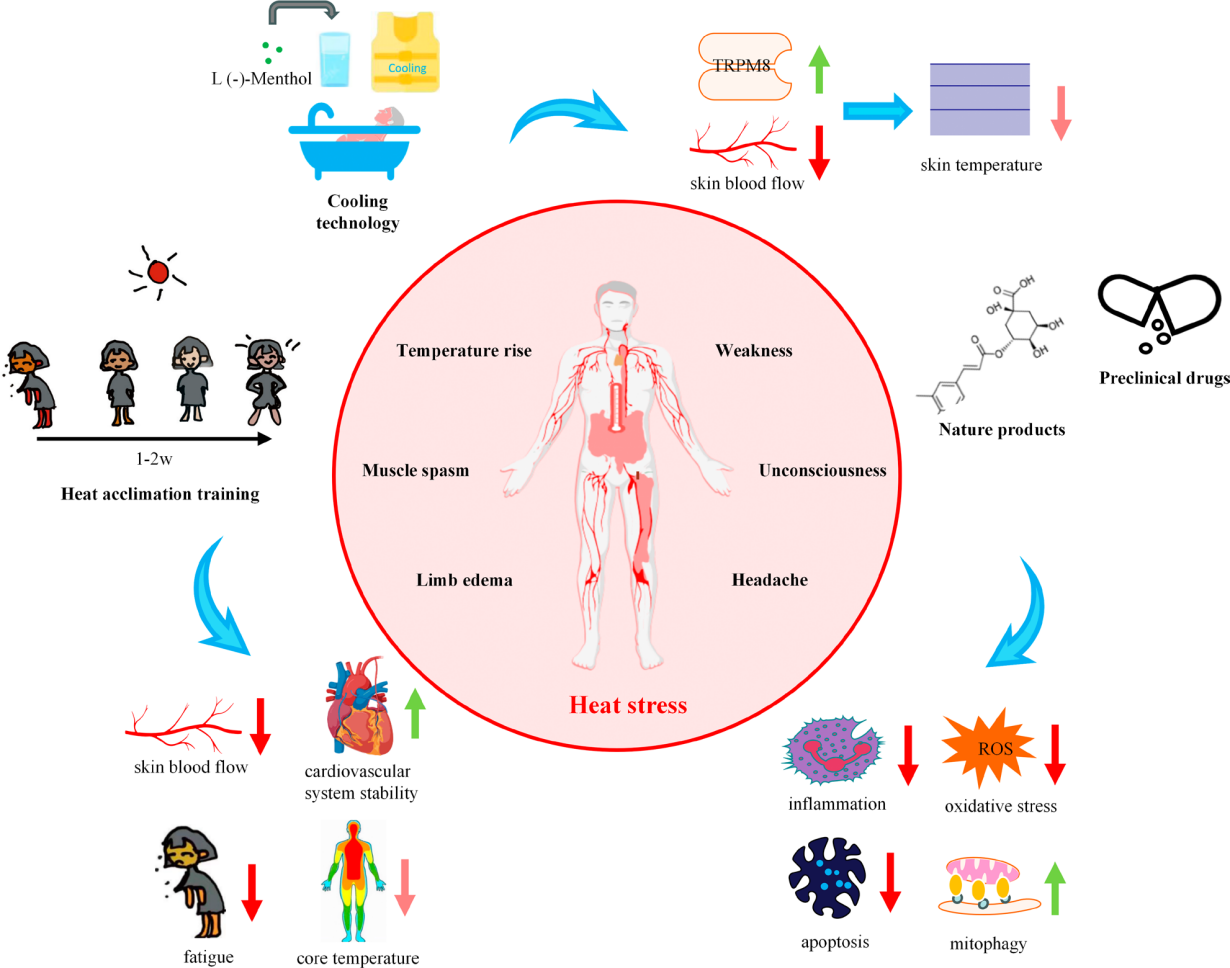
Coping strategies for heat stress. Heat stress can lead to increased body temperature, weakness, muscle cramps, limb edema, loss of consciousness, and headaches. Current coping strategies for heat stress include cooling techniques, heat acclimation training, and pharmacotherapy. Cooling techniques reduce skin temperature by activating cold receptor TRPM8 channels and reducing blood flow to the skin. Heat acclimation training reduces the body's core temperature by decreasing blood flow to the skin, promoting cardiovascular stabilization, and reducing fatigue. Natural ingredients and preclinical drugs extracted from plants can restore organ function by improving pathological processes such as inflammatory response, oxidative stress, apoptosis, and autophagy disorders of the organism under heat stress, thus reducing heat stress injury.

The body is thermoregulated through autonomic activity to maintain internal core temperature within a normal range. Basic metabolic activities generate an internal heat load, and exogenous heat in the external environment exacerbates the challenge of heat dissipation, potentially overloading the body's thermoregulatory capacity and leading to several heat‐related illnesses, including heat cramps, heat edema, heat exhaustion, and pyrexia. These can further lead to multi‐organ dysfunctions such as intestinal epithelial dysfunction, liver damage, cardiovascular system dysfunction, etc. (Sorensen and Hess 2022). The treatment of heat injury includes general therapy (e.g., cooling therapy, electrolyte supplementation, etc.), heat acclimatization, and pharmacological regimens that have been used clinically or in preclinical studies. In addition, a large number of preclinical studies are now proposing new pharmacological options to address the pathogenesis of heat stress injuries, such as inflammation, oxidative stress, and the heat shock response (Figure 8).

### 4.1 General Treatment

#### 4.1.1 Cooling Technology

Targeted temperature management (TTM) is particularly important for patients with thermal injuries, aiming to maintain rectal temperatures between 37.0°C and 38.5°C (Stanger et al. 2018). Cooling techniques can be categorized into external and internal cooling. External cooling techniques, which mainly include cooling suits, cold water immersion, or fanning, aim to reduce the degree of heat stress by increasing the temperature gradient from the core to the skin as well as improving thermal perception. Internal cooling aims to reduce the core temperature and reduce the heat load through the heat dissipation effect of cold fluids or melting ice (Ross et al. 2011; Schulze et al. 2015). Often, the first step in the treatment of patients with thermal injuries is to enhance external and internal cooling.

##### 4.1.1.1 External Cooling

External cooling is used to reduce thermal strain and slow the rise in core temperature by increasing heat transfer from the core to the periphery (Kay et al. 1999). Cooling garments such as cooling undershirts and ice undershirts usually target the trunk area, which represents 24% of the body surface area, and cold water immersion usually targets 90% of the area below the chest; this type of external cooling reduces skin temperature by decreasing blood flow to the skin. The gold standard treatment for thermal injuries has been reported to be complete cold water immersion, which rapidly reduces core body temperature (Armstrong et al. 1996). A systematic evaluation by Douma's team analyzed the effectiveness of water immersion techniques after pyrexia in 63 studies and found that the use of water at 1°C–17°C was more effective in reducing core body temperature (Douma et al. 2020). Neck and head cooling are limited by their small surface area, so cooling garments that cover these areas have little effect on core temperature (Tikuisis et al. 2001). However, cooling the neck and head reduces skin temperature and disproportionately ameliorates heat stress by stimulating thermoreceptors (Cotter and Taylor 2005).

##### 4.1.1.2 Internal Cooling

Supplemental ice slurry beverages are commonly used as a means of internal cooling and consist of millions of very small ice particles submerged in a liquid. The small particle size of ice slurries provides a greater surface area for heat transfer, and the additional energy required for the phase change from ice to water—which is three times greater than the phase change that occurs when heating cold water to average body temperature—suggests that ice can provide greater effectiveness in terms of heat dissipation. Further studies have found that the liquid from ice slurry drinks promotes heat transfer within the body, such as the mouth, esophagus, stomach, and intestines, by facilitating contact between the ice particles and the tissues of the digestive system. Ice slush drink intake directly reduces core temperature due to the energy required to heat the ingested liquid to body temperature (Tan and Lee 2015). In addition, menthol can be added to existing cold beverages as a cooling agent, which is capable of inducing a feeling of freshness, coolness, and nasal comfort in the body by activating the cold receptor TRPM8 channels (Peier et al. 2002; Mckemy et al. 2002). It was found that ingestion of a menthol‐flavored beverage significantly increased the body's resistance to heat at high temperatures (30.7°C, 78% relative humidity) compared to a control group that ingested the same beverage without the mint additive, and that menthol could therefore be used as a perceptible internal cooling strategy (Riera et al. 2014). In addition, a rapid intravenous infusion of cold saline at 4°C can also produce an effective cooling effect, particularly in patients with heat shock following dehydration, and this is often used as part of a combination of treatments. Based on the literature, the consensus recommends infusion of saline at 25 mL/kg or a total volume of 1000–1500 mL for 60 min at 4°C. The key to this approach is to maintain a rapid rate of infusion; otherwise, the cooling effect will not be achieved. Cooling should also be accompanied by monitoring of core temperature and should not drop below 38.5°C. Saline at room temperature can also be used for cooling if cold saline is not available on‐site (Liu et al. 2020).

#### 4.1.2 Heat Acclimation

Heat acclimation is an adaptive response to prolonged heat exposure. When the body is exposed to heat for a prolonged period at a certain frequency, heat stress tolerance develops, which is manifested by an increased sweating rate, decreased cutaneous blood flow rate, decreased core and skin temperatures, decreased metabolic rate, and increased circulatory and cardiovascular stability. These adjustments reduce thermoregulatory strain and the risk of heat‐related illnesses induced by high temperatures (Mortola and Frappell 2000). Heat acclimatization is a highly individualized process that depends on a variety of factors, such as the active or passive nature of heat acclimatization in an individual, the duration, frequency, and number of heat exposures, and the environmental conditions under which heat acclimatization occurs. It has been found that the acquisition of heat acclimatization occurs relatively quickly and that most of the acclimatization changes in certain physiological indices, such as the increase in plasma volume and decrease in heart rate, occur within the first week of heat exposure (Pandolf 1998). Furthermore, thermal acclimatization sessions of 2 weeks or more maximize acclimatization and associated physiological benefits (Périard et al. 2015). However, thermal acclimatization by the organism is only maintained for a short period. When removed from the thermal environment, the various acclimatization manifestations of the body decay at a rate of 2.5% per day (Daanen et al. 2018). Of note, the time or intensity of exposure to heat acclimatization has a certain limit; if the external thermal stimulus is too strong or too long, physiological disorders and a series of negative neurological responses, such as fatigue, irritability, loss of appetite, and even heat stroke, may ensue. Different heat exposure times may lead to different physiological responses in the body. The underlying mechanisms of these responses need to be explored.

In recent years, the development of information technology has brought about changes in people's lifestyles, and sedentary behavior has brought about great harm, so the call for ‘exercise is medicine ’ is growing, and scientific research has likewise focused on this. Interestingly, exercise combined with heat exposure induces physiological adaptations that reduce the risk of severe heat stroke and enhance exercise performance in thermal environments by improving heart rate, sweat rate, blood, fluid, and cellular response mechanisms, and consequently thermoregulation (Périard et al. 2015). Also in competitive sports, 10 days of passive heat‐exposure training results in improved heart rate, sweat rate, and increased sport‐specific performance in rugby players; this thermoregulatory benefit can persist beyond the end of the intervention (Fenemor et al. 2023; Fenemor et al. 2022). Active heat exposure (i.e., a thermal environment combined with exercise) improves performance and reduces fatigue (Gale et al. 2021). Thus, either periodic active or passive thermal environmental exposures can improve thermoregulatory mechanisms and enhance exercise performance, which provides more feasible measures and enables the development of heat action programs. To explore more applied benefits of exercise under heat exposure, sedentary populations have been identified to experience favorable lipid and immune profile changes and improved blood pressure and lipids during long‐term exercise at high temperatures (Rivas et al. 2019). The benefits of interventions incorporating heat exposure could provide viable intervention options for other clinical populations with vascular, metabolic, and immune dysfunctions. Therefore, heat acclimatization is beneficial not only for the prevention of single heat injuries but also for heat injuries under exercise conditions.

In terms of molecular mechanisms, the acquisition of thermal acclimatization is closely linked to related genomic responses and molecular signaling. This includes neuroplasticity of the thermoregulatory system, such as the induction of altered temperature thresholds in heat sink effectors, altered expression of protective molecules in the hypothalamus and cardiac cells, such as HSPs and hypoxia‐inducible factor‐1α, altered expression of genes involved in cross‐tolerance, and cardiac remodeling to increase contractile efficiency by inducing the myosin isoform spectrum (Horowitz 1985; Horowitz and Kodesh 2010). Specifically, prolonged heat acclimation promotes the transcription of HSPs, causing alterations in DNA methylation and post‐translational modifications of histone proteins, resulting in epigenetic adaptive changes that resist more severe heat‐exposure shocks or heat stress‐induced pathological responses, such as oxidative stress and mitochondrial damage (13).

### 4.2 Emerging Strategies

After an organism suffers heat injury, although it may be possible to normalize body temperature by various physical means, it may still be impossible to prevent the progression of pathological processes such as inflammation, oxidative stress, autophagy disorders, and multi‐organ dysfunction in the organism. For patients with early mild heat injury, oral administration of Huo Xiang Zheng Qi Liquid and topical application of herbal medicines such as wind oil or cooling oil can achieve the effect of preventing heat stroke. However, for patients with severe heat stress or even pyrexia, targeted medication or clinical first aid is needed for the damaged organs. Currently, no drugs have been clinically proven to be effective against heat stress in the organism, so the development of safe and effective pharmacological treatments for heat stress to counteract cytopathological changes such as inflammation, oxidative stress, and autophagy disorders in the organism is a top priority. A subset of preclinical drugs already exists to target heat stress injury, but further clinical trials are needed before they can be put into human therapy.

Glucocorticoids such as dexamethasone, hydrocortisone, and epinephrine are commonly used clinically to treat inflammatory responses to heat stress (Liu et al. 2020). Dexamethasone is an immunosuppressive drug used for inflammation control, and it has been reported that dexamethasone significantly reduces the expression levels of serum TNF‐α and IL‐1β in pyrexia rats, inhibits systemic inflammatory responses and blood hypercoagulability, and thereby attenuates multi‐organ damage (Liu et al. 2014). HMGB1 is a key factor in heat stress‐induced inflammation, and isoproterenol was found to inhibit the release of intracellular HMBG1 to attenuate the secretion of inflammatory factors, thereby alleviating heat stress‐induced cellular damage (Tang, Deng, et al. 2013). In addition, HMBG1 monoclonal antibody pretreatment significantly down‐regulated plasma TNF‐α, IL‐1β, IL‐6 as well as AST and ALT, in heat‐stressed rats, thereby ameliorating liver injury (Tong et al. 2013). Theanine (LTA) has significant anti‐inflammatory bioactivities, as it inhibits the heat stress‐activated P38 signaling pathway, promotes the phosphorylation of P65, which inhibits the expression of the inflammatory factors TNF‐α, IL‐6, and IL‐1β, as well as the up‐regulation of AST and ALT enzymes, and prevents the inactivation of GSH‐Px, ultimately alleviating inflammation and excessive oxidation (Liu et al. 2022; Hu et al. 2023). In a recent study by Umemura et al., a bone marrow‐derived mononuclear cell (BMMNC) transplantation therapy was found to reduce acute systemic inflammation and vascular disease in heat‐stressed rats by significantly increasing the secretion of anti‐inflammatory factors and significantly down‐regulating the expression of the pro‐inflammatory factors TNF‐alpha and IL‐6 in the lungs, kidneys, and spleens of rats injected intravenously with BMMNCs for 24 h. The results showed a significant increase in the secretion of anti‐inflammatory factors and a significant downregulation of the expression of the pro‐inflammatory factors TNF‐alpha and IL‐6, resulting in endothelial damage and multi‐organ dysfunction in heat‐stressed rats (Umemura et al. 2018). In addition, treatment with human umbilical cord blood‐derived CD34+ cells significantly suppressed the inflammatory response and promoted the expression of plasma IL‐10 and brain glial cell‐derived neurotrophic factor, thereby attenuating brain dysfunction (Chen et al. 2007).

Antioxidant therapy is also an important future direction in the treatment of heat stress injury. Treatment with Alad‐1, an aldehyde dehydrogenase 2 (ALDH2) agonist, scavenges heat stress‐induced ROS accumulation, thereby alleviating oxidative stress injury (Tsai et al. 2021). LTA is able to alleviate heat stress‐induced proteotoxic stress by inhibiting the HSF1/Hsp70 signaling axis while inhibiting HSF1 expression to promote AMPK phosphorylation, thereby inhibiting fatty acid synthesis and hepatic gluconeogenesis and ameliorating hepatic injury to alleviate heat stress‐induced metabolic stress (Lin et al. 2023). Gintin‐Enriched Fraction (GEF), a glycolipid protein derived from a non‐saponin constituent of ginseng, has been shown to protect muscle cells from heat stress‐induced oxidative stress thanks to its inhibitory effect on the p38 and ERK signaling pathways (Chei et al. 2020). In addition, dietary supplementation of chlorogenic acid activated the Nrf2/HO‐1 pathway, attenuated heat stress‐induced high expression of urea nitrogen, alanine aminotransferase, aspartate aminotransferase, cysteine‐8, and cysteine‐3, and attenuated oxidative stress and hepatocellular apoptosis induced by heat stress, which promoted the antioxidant capacity and growth performance of rabbits in a hyperthermic environment (Ji et al. 2024). Shengmisan (SMS), a Chinese herbal formula that originated in the Jin Dynasty, has been shown to have some therapeutic effects on heat stress. Zhang et al. found that SMS was able to promote the phosphorylation of AMPK and maintain mitochondrial homeostasis through the dynamin 1 (Drp1)‐dependent mitochondrial autophagy process, which suppressed the overexpression of heat shock proteins and oxidative damage in hepatic tissues following heat exposure and ultimately alleviated thermal energy metabolism under heat stress (Zhang et al. 2022).

Moreover, LTA intervention also increases the concentration of amino acids and lipid metabolites, modulates gut microbiota and metabolite‐stimulated macrophage differentiation, reduces macrophage antigen presentation to the specific immune system, promotes B‐cell differentiation and sIgA secretion, inhibits pro‐inflammatory factors, and enhances intestinal defenses, thereby alleviating pyrexia in rats (Liu et al. 2024). Hydroxysafflor yellow A (HSYA) is an active natural ingredient extracted from the flowers of *Carthamus tinctorius* L. HSYA was able to reduce p38 and Hsp27‐78 phosphorylation levels and MK‐2 expression to promote autophagy, increase Bcl‐2 expression, and inhibit Bax and caspase‐3 expression in neural stem cells under heat stress thus, ameliorating apoptosis caused by heat stress (Li, Liu, et al. 2019). Although there are a large number of basic or preclinical studies confirming the possibility of targeted drug therapy for heat stress injury, the real application of these drugs to the treatment of clinical heat injury diseases still requires a long time of clinical exploration and validation.

In summary, recovery strategies for damage caused by heat stress are usually based on traditional or classical cooling methods, such as internal and external cooling techniques. However, it is more important to understand that long‐term thermal acclimatization improves heat tolerance as it is more reliable and independent. Unfortunately, there are few studies on the regulation of cellular physiological processes by heat acclimatization, mainly in terms of oxidative stress and mitochondrial function. In addition, pathological processes such as inflammation, oxidative stress, and impaired autophagy associated with thermal injury are key to recovery from thermal injury, but these pathological processes often cannot be fully recovered by physical means. To further explore safe and effective modalities, current research perspectives in the field are focused on natural plant extracts, especially when natural extracts can be used as dietary supplements, which may be a more attractive and convenient property.

## 5 Conclusions and Perspectives

In this review, we first discuss the thermal signaling response and a series of physiological and pathological changes such as oxidative stress, autophagy, apoptosis, etc. in cells under hyperthermia. Considering that pathological changes in these physiological responses may be a key cause of multi‐organ damage under heat stress, but there is no review that elucidates the mechanisms involved, we next focus on the physiopathological mechanisms of multi‐organ damage under heat stress. We found that heat stress causes inflammation, oxidative stress, and mitochondrial damage in multiple organ cells, and these severe pathological changes lead to organ cell degeneration and death, resulting in multiple organ injuries, such as neurological dysfunction, liver injury, cardiovascular dysfunction, intestinal dysfunction, and skeletal muscle injury. Interestingly, during heat stress, certain protective mechanisms still exist in the organism, and one of the most important protective changes is the elevated expression of HSPs, which protects the cells from heat stress injury to some extent. However, the protective effect of HSP‐mediated heat shock response exists in a certain range, and exceeding the protective range of HSPs will still lead to organ damage. In addition, most of the current studies have focused on the animal level, and further exploration of the changes in the deeper mechanisms of the human body under heat stress is needed in the future. Finally, we summarize the existing strategies for heat stress injury in the body and the related preventive measures that may be realized in the future, so as to provide more diagnostic and therapeutic ideas for the prevention and treatment of heat injury. Currently, the most classical strategy is cooling therapy, e.g., internal and external cooling techniques. However, it is more important to understand that long‐term thermal acclimatization improves heat resistance. Heat stress induced by a single thermal stimulus and heat acclimatization induced by prolonged exposure to high temperatures have different effects on the body's biological responses. Thermal acclimatization is the process of adapting to prolonged exposure to high temperatures, and the results are usually beneficial. Unfortunately, little research has been done on the regulation of cellular physiological processes by heat acclimatization, mainly in terms of oxidative stress and mitochondrial function. However, increasing heat tolerance through adaptation is a more reliable and independent way to prevent subsequent thermal damage. Therefore, heat adaptation mechanisms should be further explored to provide a theoretical basis for improving heat tolerance and athletic performance in high‐temperature environments. In addition, pathological processes such as inflammation, oxidative stress, and mitochondrial damage associated with heat injury are key to the body's recovery from heat injury, but these pathological processes are often not fully recovered by physical means, and more targeted therapeutic measures are needed. A subset of preclinical drugs already exists for the targeted treatment of multi‐organ damage in response to heat stress, but further clinical trials are needed before they can be put into human therapy.

## Author Contributions

Xiaoqing Ding wrote the manuscript and prepared the figures, and revised the manuscript. Binghong Gao (corresponding author) conceived and coordinated the work. All the authors read and approved the final manuscript.

## Ethics Statement

This review does not address the ethics associated with human or animal experimentation.

## Consent

The authors have nothing to report.

## Conflicts of Interest

The authors declare no conflicts of interest.

## Data Availability

Data sharing is not applicable to this article as no new data were created or analyzed in this study.
